# Inflammation-induced lysosomal dysfunction in human iPSC-derived microglia is exacerbated by APOE 4/4 genotype

**DOI:** 10.1186/s12974-025-03470-y

**Published:** 2025-06-02

**Authors:** Marianna Hellén, Isabelle Weert, Stephan A. Müller, Noora Räsänen, Pinja Kettunen, Šárka Lehtonen, Michael Peitz, Klaus Fließbach, Mari Takalo, Marja Koskuvi, Stefan F. Lichtenthaler, Ville Leinonen, Alfredo Ramirez, Olli Kärkkäinen, Mikko Hiltunen, Jari Koistinaho, Taisia Rõlova

**Affiliations:** 1 Neuroscience Center, Helsinki Institute of Life Science (HiLIFE), University of Helsinki, Helsinki, Finland; 2 German Center for Neurodegenerative Diseases (DZNE), Munich, Germany; 3 Neuroproteomics, School of Medicine and Health, Klinikum rechts der Isar, Technical University of Munich, Munich, Germany; 4 A. I. Virtanen Institute for Molecular Sciences, University of Eastern Finland, Kuopio, Finland; 5 Institute of Reconstructive Neurobiology, University of Bonn Medical Faculty & University Hospital Bonn, Bonn, Germany; 6 Cell Programming Core Facility, University of Bonn Medical Faculty, Bonn, Germany; 7 Department of Old Age Psychiatry and Cognitive Disorders, University of Bonn Medical Center, Bonn, Germany; 8 German Center for Neurodegenerative Diseases (DZNE Bonn), Bonn, Germany; 9 Institute of Biomedicine, University of Eastern Finland, Kuopio, Finland; 10 Munich Cluster for Systems Neurology (SyNergy), Munich, Germany; 11 Department of Neurosurgery, Kuopio University Hospital, Kuopio, Finland; 12 Institute of Clinical Medicine – Neurosurgery, University of Eastern Finland, Kuopio, Finland; 13 Division of Neurogenetics and Molecular Psychiatry, Department of Psychiatry and Psychotherapy, Medical Faculty, University of Cologne, Cologne, Germany; 14 Department of Old Age Psychiatry and Cognitive Disorders, University Hospital Bonn, University of Bonn, Bonn, Germany; 15 Glenn Biggs Institute for Alzheimer’s & Neurodegenerative Diseases, University of Texas Health Sciences Center, San Antonio, TX USA; 16 Cologne Excellence Cluster on Cellular Stress Responses in Aging-Associated Disease (CECAD), University of Cologne, Cologne, Germany; 17 Afekta Technologies Ltd, Kuopio, Finland; 18 School of Pharmacy, University of Eastern Finland, Kuopio, Finland; 19 Drug Research Program, Division of Pharmacology and Pharmacotherapy, University of Helsinki, Helsinki, Finland

**Keywords:** Microglia, Alzheimer’s disease, Apolipoprotein E, iPSC, Lysosomal dysfunction

## Abstract

**Background:**

The ε4 isoform of apolipoprotein E (ApoE) is the most significant genetic risk factor for Alzheimer’s disease. Glial cells are the main source of ApoE in the brain, and in microglia, the ε4 isoform of ApoE has been shown to impair mitochondrial metabolism and the uptake of lipids and Aβ42. However, whether the ε4 isoform alters autophagy or lysosomal activity in microglia in basal and inflammatory conditions is unknown.

**Methods:**

Altogether, microglia-like cells (iMGs) from eight *APOE*3/3 and six *APOE*4/4 human induced pluripotent stem cell (iPSC) lines were used in this study. The responses of iMGs to Aβ42, LPS and IFNγ were studied by metabolomics, proteomics, and functional assays.

**Results:**

Here, we demonstrate that iMGs with the *APOE*4/4 genotype exhibit reduced basal pinocytosis levels compared to *APOE*3/3 iMGs. Inflammatory stimulation with a combination of LPS and IFNγ or Aβ42 induced PI3K/AKT/mTORC signaling pathway, increased pinocytosis, and blocked autophagic flux, leading to the accumulation of sequestosome 1 (p62) in both *APOE*4/4 and *APOE*3/3 iMGs. Exposure to Aβ42 furthermore caused lysosomal membrane permeabilization, which was significantly stronger in *APOE*4/4 iMGs and positively correlated with the secretion of the proinflammatory chemokine IL-8. Metabolomics analysis indicated a dysregulation in amino acid metabolism, primarily L-glutamine, in *APOE*4/4 iMGs.

**Conclusions:**

Overall, our results suggest that inflammation-induced metabolic reprogramming places lysosomes under substantial stress. Lysosomal stress is more detrimental in *APOE*4/4 microglia, which exhibit endo-lysosomal defects.

**Supplementary Information:**

The online version contains supplementary material available at 10.1186/s12974-025-03470-y.

## Background

Lysosomal dysfunction is a common feature of aging-associated neurodegenerative diseases, including Alzheimer’s disease (AD), which is characterized by the accumulation of toxic beta-amyloid (Aβ) and tau protein aggregates in the brain [1]. Since autophagy and endocytosis/phagocytosis pathways deliver dysfunctional organelles, extracellular material, and debris to lysosomes for degradation, lysosomal dysfunction has been suggested as a key factor promoting the accumulation of dysfunctional organelles and protein aggregates in AD [2].

Lysosome biogenesis is controlled by the microphthalmia-associated transcription factor/transcription factor E (MIT/TFE) family of transcription factors, including TFEB, TFE3, MITF, and TFEC [3]. These transcription factors bind to Coordinated Lysosomal Expression and Regulation (CLEAR) motifs and co-operate to fine-tune lysosomal gene expression in various conditions [4]. Disruption of TFEB-mediated signaling has been reported to exacerbate tau pathology [4, 5], while TFEB overexpression in neurons and astrocytes may enhance the clearance of Aβ and tau in mouse models of AD [6–8], highlighting a strong link between lysosomal dysfunction and AD pathology.

Apolipoprotein E (ApoE) is the primary carrier of cholesterol and triglycerides in the bloodstream. In the brain, it is primarily produced by astrocytes [9] and disease-associated microglia [10]. Among the three human isoforms (ε2, ε3, and ε4), ApoE ε3 (further referred to as E3) is considered neutral, while ApoE ε4 (henceforth referred to as E4) represents the most significant genetic risk factor for AD [11–13]. It is estimated that up to 50% of AD patients carry at least one E4 allele [12].

E4 profoundly alters lipid metabolism in various cell types [14–18] and impairs autophagy and lysosomal activity in astrocytes and neurons [19–23]. In fibroblasts, E4 may directly interfere with TFEB binding to CLEAR motifs, thereby decreasing the transcription of lysosomal genes [24].

Microglia, the immune cells of the brain, play a critical role in AD pathogenesis by regulating the clearance, deposition, and propagation of Aβ and tau aggregates, as well as mounting the inflammatory response [25–27]. Previous studies have reported that, compared to human E3/E3 homozygous microglia, E4/E4 microglia exhibit significant transcriptional alterations, impaired uptake of lipids and Aβ, and deficits in mitochondrial metabolism, calcium signaling, and migration [14–16, 28–30]. However, whether the E4 variant alters autophagy or lysosomal activity in microglia in basal or inflammatory conditions is unknown.

To address this question, we utilized human induced pluripotent stem cell (iPSC)-derived microglia (iMGs) with E4/E4 and E3/E3 genotypes to investigate whether E4/E4, as a significant genetic risk factor for AD, compromises microglial endocytosis-lysosome pathways or autophagy in homeostatic or inflammatory conditions. Our study provides evidence that the E4/E4 genotype impairs pinocytosis and lysosomal activity via the mammalian target of rapamycin (mTORC)1 pathway in both homeostatic and activated iMGs compared to the E3/E3 genotype. In contrast, autophagy appears unaffected by the *APOE* genotype.

## Materials and methods

### Differentiation of human iPSC-derived microglia (iMGs)

The iPSC lines used in this study are listed in Supplementary materials, Table 1. Human iPSC lines were maintained in Essential 8 medium (Thermo Fisher Scientific) on Matrigel (growth factor reduced; Corning; 1:200)-coated 3.5 cm dishes at 37 °C and 5% CO2. The cells were passaged with 0.5 mM EDTA every 4–5 days. The iMGs were differentiated from iPSCs as described previously [31–33]. In brief, iPSC colonies were detached using ReLeSR reagent (STEMCELL Technologies), plated at density of 3–6 colonies per cm^2^ on Matrigel-coated 6-well plates (1:200) in Essential 8 medium supplemented with 5 µM ROCK inhibitor Y-27,632 (Selleckchem), and differentiated into hematopoietic progenitors using the commercial STEMdiff Hematopoietic kit (STEMCELL Technologies) for 11–13 days. Floating hematopoietic progenitors were then collected and plated at a density of 7000–8000 cells per cm^2^ on new Matrigel-coated 6-well plates. The cells were grown for 27 days in microglial differentiation medium containing DMEM/F12, 2× insulin-transferrin-selenite, 2× B27, 0.5× N2, 1× Glutamax, 1× non-essential amino acids (all from Thermo Fisher Scientific), 400 µM monothioglycerol (Merck Millipore), 5 µg/mL human insulin (Merck Millipore), 100 ng/mL human interleukin-34 (IL-34) (Sino Biological), 50 ng/mL human transforming growth factor beta 1 (TGF-β1), and 25 ng/mL human macrophage colony-stimulating factor (M-CSF) (Peprotech, Thermo Fisher Scientific). Fresh medium was added every other day. To promote microglial maturation, 100 ng/mL human CD200 (Biolegend) and 100 ng/mL human C-X3-C motif chemokine ligand 1 (CX3CL1) (Peprotech, Thermo Fisher Scientific) were added to the cells during the last 4 days of culture. The generated cells exhibited immunopositivity for the microglial markers Iba1 (ionized calcium binding adaptor molecule 1), CD18 (β2 integrin), CX3CR1 (C-X3-C motif chemokine receptor 1), and TREM2 (triggering receptor expressed on myeloid cells 2) (Supplementary Fig. 2A).

### LPS and LPS plus IFNγ treatment

The iMGs were replated at the density of 50,000 to 70,000 cells per cm^2^ 4–5 days before the experiment in the maturation medium. The cells were then treated with 100 ng/ml LPS (Merck Millipore) alone or with the combination of 50 ng/ml LPS and 15 ng/ml human IFNγ (Peprotech, Thermo Fisher Scientific) for 24–48 h in iMG medium containing only IL-34 and M-CSF but no other cytokines.

### Soluble Aβ42 treatment

Human Aβ (1–42 trifluoroacetate, Bachem) was solubilized completely using 1,1,1,3,3,3-Hexafluoro-2-propanol (HIFP). After complete evaporation of HIFP using SpeedVac Vacuum concentrator, Aβ was reconstituted in dimethyl sulfoxide (DMSO) at a final concentration of 2.5 mM and sonicated for 10 min in the water bath sonicator.

Then Aβ was diluted to 100 µM concentration in cold phosphate-buffered saline Dulbecco’s phosphate-buffered saline (D-PBS; Thermo Fisher Scientific) and incubated for three days at RT. The endotoxin concentration (48 EU/ml) of the 100 µM Aβ42 was quantitatively measured by using ToxinSensorTM Chromogenic LAL Endotoxin Assay Kit (GenScript, Cat L00350C) according to the manufacturer’s instructions. Then Aβ42 was subsequently added to the cells at final concentration of 200 nM and incubated for 48 h at 37 °C before harvesting the cells and media.

For Western blot (WB), the iMGs were maturated on Matrigel-coated (1:100) 6-well plates at density of 47,000 cells per cm^2^ for 4 days. The treatment was started 48 h before collection by replacing half of the old media with fresh iMG medium containing only IL-34 and M-CSF cytokines with or without soluble Aβ42 oligomers. For one E4/E4 cell line, rapamycin was given at a final concentration of 100 nM 24 h before collection. On a day of collection, bafilomycin-treated cells were incubated for 3 h at 37 °C with bafilomycin A1 (Lysosomal Activity Assay Kit ab234622, Abcam), diluted according to the manufacturer’s instructions) before lysing the cells.

### L-leucyl-L-leucine Methyl ester (LLOMe) treatment

For studying the lysosomal membrane permeabilization by the LLOMe treatment assay, the cells were plated at a density of 45,500 cells per cm^2^ onto coverslips coated with Matrigel (1:100). On the treatment day, H-Leu-Leu-OMe Hydrochloride (LLOMe) (#6491-83-4, Santa Cruz Biotechnology) was dissolved in DMSO, old media was removed from the cells and 250 µl of fresh iMG medium without cytokines supplemented with DMSO (vehicle) or 200 µM LLOME was added on top of the cells. The iMGs were incubated for 2 h at 37 °C before fixation with 4% formaldehyde in D-PBS supplemented with 0.9 mM CaCl_2_ and 0.5 mM MgCl_2_ at RT for 20 min.

### Immunocytochemistry

The iMGs were permeabilized and unspecific binding sites blocked with 0.3% Triton X-100 in 5% normal goat serum in D-PBS at RT for 1 h. The iMGs were incubated with primary antibody (Supplementary materials, Table 2) in 5% normal goat serum in D-PBS at 4 °C overnight following secondary antibody incubation (Supplementary materials, Table 2) at RT for 1 h. Nuclei were visualized by DAPI (Merck Millipore) staining at RT for 5 min and the coverslips were mounted with Fluoromount-G Mounting Medium (Thermo Fisher Scientific). LGALS1 primary antibody was a generous gift from Prof. P. Laakkonen, University of Helsinki. The images of LGALS1-stained cells were acquired with Zeiss LSM980 confocal microscope with C-Apochromat 63x/1.20 W Korr UV VIS IR objective. LGALS1 puncta were quantified with Fiji ImageJ v. 1.53 software using the Gaussian blur filter and the difference of Gaussians. The number of puncta in the image was normalized by the number of cell nuclei. Three images per iPSC line per treatment were quantified.

### Reverse transcription quantitative real-time PCR (RT-qPCR) for in vitro iMGs

RNA was isolated by using Qiagen RNeasy Mini Kit (#74106, Qiagen) according to manufacturer’s instructions. cDNA was synthetized from isolated RNA by using Maxima reverse transcriptase enzyme (#EP0742, Thermo Fisher Scientific) according to manufacturer’s instructions. The expression levels of genes of interest were measured by using Maxima Probe/ROX qPCR Master Mix (#11813923, Thermo Fisher Scientific) and the Taqman primers listed in Supplementary materials, Table 3 on Bio-Rad CFX96 Real-Time System (Bio-Rad). The relative mRNA expression results were normalized to the ΔC_T_ averages of two housekeeping genes *GAPDH* and *ACTB* using 2^−ΔΔCT^ method.

### Cell Mito Stress mitochondrial function assay

The Seahorse XF Cell Mito Stress Test (Agilent) was used to measure the key parameters of the mitochondrial function in the cells. The manufacturer’s instructions were followed for the workflow of the experiment. Briefly, the cells were plated 40,000 cells per well in 200 µl maturation medium one week before the experiment, and half of the medium was replaced with a fresh medium every other day. On the day of the experiment, the Seahorse XF Assay medium was prepared by supplementing the Seahorse DMEM medium with Glutamax (Gibco, Thermo Fisher Scientific) to a final concentration of 2 mM. The cells were rinsed with 180 µl of Seahorse XF medium followed by the addition of Seahorse XF medium to a final volume of 180 µl. The cells were incubated in a non-CO_2_ incubator at 37 °C for 1 h before running on the XFe96 Analyzer (Agilent). At the beginning of the assay, glucose and sodium pyruvate (Gibco) were added by the XFe96 Analyzer to cells to final concentrations of 10 mM and 1 mM, respectively. Next, modulators of the electron transport chain all 1 µM were injected in the following order: oligomycin (Cayman Chemical) to inhibit ATP synthase and determine ATP production of the cells, Carbonyl cyanide-4 (trifluoromethoxy) phenylhydrazone (FCCP) (Cayman Chemical) to collapse the proton gradient and disrupt the mitochondrial membrane potential and to determine the maximal and spare respiratory capacity of the cells, a mixture of rotenone (Cayman Chemical) and antimycin a (Merck Millipore) to inhibit complexes I and III and to determine nonmitochondrial respiration of the cells. Oxygen consumption rate (OCR) was directly measured by XFe96 Analyzer during the assay. Results were normalized by the cell confluence analyzed by IncuCyte S3 at the Biomedicum Stem Cell Center, University of Helsinki, before the beginning of the assay and the key parameters of the mitochondrial function of the cells were calculated using Seahorse Wave Software and exported to Excel.

### Glycolysis stress test

The Agilent Seahorse XF Glycolysis Stress Test was used to measure the glycolytic function of the cells, and it was performed simultaneously with the Agilent Seahorse XF Cell Mito Stress Test. Extracellular acidification rate (ECAR) was directly measured by the XFe96 Analyzer during the assay. The manufacturer’s instructions were followed for the workflow of the experiment. First, glucose and sodium pyruvate were injected by XFe96 Analyzer on cells to final concentrations of 10 mM and 1 mM, respectively. Glucose was partially catabolized by the cells through the glycolytic pathway to pyruvate, leading to a rapid increase in ECAR, which was reported as glycolysis under basal conditions. Next oligomycin was injected on cells to inhibit ATP synthase and to shift the energy production to glycolysis. The following increase in ECAR was used to determine the cellular maximum glycolytic capacity. The obtained ECAR values were normalized to the cell confluence assessed by IncuCyte S3, and the key parameters of the glycolytic function of the cells were calculated using Seahorse Wave Software and exported to Excel.

### Cytokine quantification

Media were collected and centrifuged at 16,000 G 10 min 4 °C to remove debris. The cells were lysed using RIPA Lysis and Extraction Buffer (#89900) supplemented with protease (#A32955) and phosphatase (#A32957) inhibitors (all from Thermo Fisher Scientific). The protein concentration was measured using BCA kit (#10741395, Thermo Fisher Scientific). The media and lysates were stored at -80 °C until analysis.

The levels of human TNFα (tumor necrosis factor alpha), CCL5 (C-C motif chemokine ligand 5), CCL3 (C-C motif chemokine ligand 5), IL6 (interleukin-6), and IL8 (interleukin-8) in the media, were measured using the mixture of corresponding Cytometric Bead Array (CBA) Flex sets and Human Soluble Protein Master Buffer Kit (both from BD Biosciences) according to manufacturer’s instructions. The samples were analyzed using the BD Accuri C6 Plus flow cytometer with BD CSampler Plus software (BD Biosciences) at the Biomedicum Flow Cytometry Core Facility, University of Helsinki. Mean PE-Height fluorescence intensity values were used to construct the standard curves. Concentration values were derived from standard curves using log-log regression and normalized to protein concentration in cell lysates.

### Western blot

To study the autophagic flux in basal conditions, the iMGs were maturated on Matrigel-coated (1:200) 12-well plates at 200,000 cells per well for 5 days. On the day of the collection, the cells were treated with either rapamycin only at a final concentration of 100 nM for 6 h, bafilomycin A1 only (Lysosomal Activity Assay Kit ab234622, Abcam, diluted according to the manufacturer’s instructions) for 3 h or a combination of rapamycin (100 nM) and bafilomycin A1 for 6 h before collecting the cells.

The cells were lysed with RIPA Lysis and Extraction Buffer (#89900) supplemented with protease (#A32955) and phosphatase (#A32957) inhibitors (all from Thermo Fisher Scientific). The protein concentration was measured using BCA kit (#10741395, Thermo Fisher Scientific) and the proteins were denatured by boiling in 4xSample Buffer (62.5mM Tris-HCl pH6.8, 2.5% SDS, 0.002% Bromophenol blue, 5% β-mercaptoethanol and 10% glycerol). Total amount of 5–10 µg of protein were loaded and separated on a 4–20% Mini-PROTEAN^®^ TGX™ Gels (#4561094, Bio-Rad) and transferred to polyvinylidene fluoride (PVDF) membranes using the Trans-Blot Turbo Transfer system (Bio-Rad). The membranes were blocked in 5% fat-free milk in TBS-0.05% Tween (TBST) buffer at RT for 1 h and incubated overnight with the primary antibody diluted in 5% bovine serum albumin – 0,02% Na azide in TBST at 4 °C. The following day the membranes were washed and incubated with the horseradish peroxidase (HRP)-conjugated secondary antibodies for 1 h at RT. After washes the chemiluminescence signal was detected by using ECL Plus (#32132) or ECL (#32106) Western Blotting Substrate (both Thermo Fisher Scientific) and G: BOX Chemi XX6 (Syngene) imaging system. ImageJ software was used for semi-quantitive analysis of the membranes.

### Lysosomal activity

For assaying lysosomal activity, we used the Lysosomal Intracellular Activity Assay Kit (#ab234622, Abcam) and the assay was conducted according to the protocol provided by the manufacturer. Briefly, the iMGs were plated on 12-well plates at a density of 57,000 iMGs per cm^2^ and maturation was started 3–5 days prior to the experiment. One day before the experiment half of the media was replaced with iMG medium containing only IL-34 and M-CSF cytokines. On the day of the experiment the control cells and the cells to be treated with bafilomycin A1 were pelleted by centrifuging the 12-well plates at 300xg for 5 min at RT. After centrifugation the old media was removed and replaced by fresh media with or without bafilomycin A1 and the cells were incubated 1 h at 37 °C with 5% CO_2_. After incubation the cells were pelleted as above and after centrifugation the old media was removed and 500 µl of new media supplemented with Self-Quenched Substrate provided by the kit (15 µl per 1 ml of media) with or without bafilomycin A1 was added on top of the cells. The cells were then incubated 1 h at 37 °C, 5% CO_2,_ before removing all the media and adding 1 ml of ice-cold 1% bovine serum albumin in D-PBS. The cells were scraped off, washed with ice-cold 1xAssay buffer and resuspended to 500 µl of D-PBS. The mean fluorescence intensity (MFI) in FITC channel was analyzed using the BD Accuri C6 Plus flow cytometer.

### Endocytosis

For pHrodo dextran endocytosis assays, the cells were plated at a density of 62,500 cells per cm^2^ into a black-walled 96-well imaging plate in 100 µl iMG medium containing the maturation factors (CD200 and CX3CL1) 2–3 days before performing the assay. One day before the experiment half of the media was removed and 50 µl of fresh media containing treatments (final concentrations 50 ng/ml LPS and 15 ng/ml IFNγ) were added on top of cells. On a day of the assay, Invitrogen^TM^pHrodo™ Red (#10361) or Green (#P35368) 10 kDa Dextran conjugates were dissolved in D-PBS in a final concentration of 0.1 mg/ml. After removing 20 µl of old media from the wells, added 20 µl of conjugate suspension on top of the cells to a final concentration of 16,7–20 µg/ml. The endocytosis assay was performed using IncuCyte S3 live cell imaging system (Sartorius) at the Biomedicum Stem Cell Center core facility, University of Helsinki. The cells were imaged first every 30 min and after 4.5 h every 1 h for 20 h and the integrated intensity of the fluorescence signal was normalized to cell confluence assessed by IncuCyte S3 before adding the pHrodo dextran conjugates. In case of apilimod treatment, the treatments for the control and apilimod treated cells were added on the day of the assay by removing 20 µl of the old media and adding 20 µl of fresh media with DMSO as a vehicle or apilimod in a final concentration of 0.683 µM before the addition of the conjugate suspension on top of the cells.

### Non-targeted metabolomics

*Sample preparation.* Non-targeted metabolomics analysis was run by the company Afekta Technologies, Kuopio, Finland (www.afekta.com). The cell pellets, containing 560,000 to 600,000 cells each, were dissociated with 60 µl of Milli-Q water at RT. The suspension was sonicated for 5 min at RT to homogenize the cells, after which 240 µl of cold 80% aqueous methanol was immediately added to stop any remaining cellular activity and to extract the metabolites. The samples were then vortexed for 10 s at RT and let settle down for 5–10 min. The centrifugation was performed at 13 000 rpm and 4 °C for 5 min. Immediately after centrifugation, the supernatant was collected carefully without disturbing the pellet with a 1 ml syringe and injected into an HPLC vial with a glass insert. The samples were stored at − 20 °C until analysis.

*LC–MS analysis.* The samples were analyzed by liquid chromatography–mass spectrometry, consisting of a Vanquish Flex UHPLC system (Thermo Fisher Scientific) coupled with a high-resolution Orbitrap mass spectrometer (Q Exactive Focus, Thermo Fisher Scientific) located in the Biocenter Kuopio, University of Eastern Finland. The analytical method has been described in detail previously [34, 35]. In brief, a Zorbax Eclipse XDB-C18 column (2.1 × 100 mm, 1.8 μm; Agilent Technologies) was used for the reversed-phase (RP) separation and an Aqcuity UPLC BEH amide column (Waters) for the hydrophilic interaction chromatography (HILIC) separation. After each chromatographic run, the ionization was carried out using jet stream electrospray ionization (ESI) in the positive and negative mode, yielding four data files per sample. The collision energies for the MS/MS analysis were selected as 10, 20 and 40 V, for compatibility with spectral databases.

*Data analysis.* Peak detection and alignment were performed in MS-DIAL ver. 4.60 [36]. For the peak collection, *m/z* values between 50 and 1500 and all retention times were considered. The amplitude of the minimum peak height was set at 120,000. The peaks were detected using the linear weighted moving average algorithm. For the alignment of the peaks across samples, the retention time tolerance was 0.05 min, and the *m/z* tolerance was 0.015 Da.

The differential features between the genotypes were detected using featurewise linear mixed models, where feature levels were predicted by genotype and cell ID was used as a random effect. The mixed models were fitted separately for samples in each of the two treatments. Benjamini–Hochberg false discovery rate (FDR) correction was performed on the p-values to account for multiple testing with results shown as q-values. All analyses were conducted with R version 3.6.3 and notame version 0.0.6.

*Compound identification.* The chromatographic and mass spectrometric characteristics (retention time, exact mass, and MS/MS spectra) of the significantly differential molecular features were compared with entries in an in-house standard library and publicly available databases, such as METLIN and HMDB, as well as with published literature. For molecular features without a match in publicly available spectral databases, a secondary annotation attempt was performed in MS-FINDER software [37] by calculating the molecular formula based on the isotopic pattern and exact mass and comparing the experimental MS/MS (if available) with in silico MS/MS spectra generated from databases of known natural and other compounds.

### Proteomics

The cells were maturated on original 6-well plates for 4 days prior to the experiment. Further, the cells were treated for 48 h with 50 ng/ml LPS and 15 ng/ml IFNγ in iMG medium supplemented only with IL-34 and M-CSF or left untreated. Then the cells were washed with 1 ml of ice-cold D-PBS and harvested by scraping and centrifuging at 1000 G for 5 min at 4 °C. The cells were then washed one more time with ice-cold D-PBS, and pellets were frozen on dry ice and kept at -80 °C until analysis.

Samples were lysed in STET lysis buffer (1% (v/v) Triton X-100, 150 mM NaCl, 2 mM EDTA, 50 mM TrisHCl pH 7.5). Cell debris and undissolved material was removed by centrifugation at 16,000 g at 4 °C for 10 min. The protein concentration was estimated using the Pierce 660 nm assay (Thermo Fisher Scientific). A modified protocol for single-pot solid-phase enhanced sample preparation (SP3) was applied. In brief, 15 µg of protein lysate MgCl_2_ was added to a final concentration of 10 mM. DNA was digested using 25 U of Benzonase (Sigma-Aldrich) per sample at 37 °C for 30 min. Proteins were reduced by adding dithiothreitol (Biozol) to a final concentration of 10 mM, followed by incubation for 30 min at 37 °C. For cysteine alkylation, iodoacetamide (Sigma-Aldrich) was added to a final concentration of 40 mM and samples were incubated 30 min at roo temperature in the dark. The reaction was quenched with an additional dose of dithiothreitol. Proteins were bound to 200 µg of a 1:1 mixture of hydrophilic and hydrophobic magnetic Sera-Mag SpeedBeads (Cytiva) by adding ethanol (Sigma-Aldrich) to a final concentration of 80% (v/v) and mixing on a thermomixer (Eppendorf) for 30 min at RT. The beads were washed four times with 200 µL of 80% ethanol using a Dynamag-2 magnetic rack (Thermo Fisher Scientific). For proteolytic digestion, 190 ng of LysC and 190 ng of trypsin (Promega) were added in 40 µL of 50 mM ammonium bicarbonate, followed by 16 h of incubation at RT. The supernatants were filtered using 0.22 μm spin-filters (Costar Spin-X, Corning) and then dried via vacuum centrifugation. The dried peptides were re-dissolved in 20 µL of 0.1% formic acid. The peptide concentration after digestion was quantified using the Qubit protein assay (Thermo Fisher Scientific). An amount of 350 ng of peptides per sample were subjected to the LC-MS/MS proteomic analyses on a nanoElute system (Bruker Daltonics) which was online coupled with a timsTOF pro mass spectrometer (Bruker Daltonics) equipped with an column oven. An amount of 350 ng of Peptides were separated on a 15 cm (75 μm ID) column self-packed with ReproSil-Pur 120 C18-AQ resin (1.9 μm, Dr. Maisch GmbH) using a 120 min long binary gradient of water and acetonitrile supplemented with 0.1% formic acid at a flow rate of 300 nL/min and a column temperature of 50 °C.

Data independent acquisition Parallel Accumulation Serial Fragmentation (DIA-PASEF) was used. One MS1 full scan was followed by 34 sequential DIA windows with 26 m/z width for peptide fragment ion spectra with an overlap of 1 m/z covering a scan range of 350 to 1200 m/z. The ramp time was fixed to 100 ms and 2 windows were scanned per ramp resulting in a total cycle time of 1.9 s. For protein label free quantification (LFQ), the LC-MS/MS raw data was analysed with the software DIA-NN [38] (version 1.8) using a library-free search against a canonical one-protein gene database of Homo sapiens from UniProt (download: 2022-01-12, 20600 entries) supplemented with a contaminants database from Maxquant (240 entries) [39]. Trypsin was defined as protease and maximum 2 missed cleavages were allowed. Acetylation of protein N-termini and methionine oxidation were defined as variable modifications whereas carbamidomethylation of cysteines was defined as fixed modification. Tolerances for mass accuracy and ion mobility were automatically optimized by DIA-NN. The false discovery rates for precursors and proteins were set to 1%.

For statistical analysis, the software Perseus (v 1.6.2.3) [40] was used. Protein LFQ values were accepted on the basis of at least 2 peptides. Contaminants were removed and protein LFQ were log2 transformed. A Student’s ttest was applied between the different groups for statistical testing of abundance differences. A permutation-based FDR correction for multiple hypotheses was applied with a p-value of 0.05 and s0 of 0.1 [41]. The FDR thresholds are visualized as hyperbolic curves.

The differentially expressed protein data that met the *p*-value < 0.05 and fold change ≥ 2 (in both directions) cutoffs were further analyzed using QIAGEN Ingenuity Pathway Analysis (IPA) as described [42].

### Statistics

Statistical analysis was done using GraphPad Prism 9.2.0 (Insight Partners, New York, NY, USA) using Student’s t-test, Mann-Whitney non-parametric test or repeated measures two-way ANOVA, with Šidák’s-corrected posthoc tests. Statistical significance was assumed at *p* < 0.05.

## Results

### Basal levels of lipidated LC3 and macropinocytosis are reduced in E4/E4 iMGs

To generate human iMGs, we used a protocol adapted from previously published studies [31, 32]. E3/E3 and E4/E4 iMGs expressed similar levels of microglial marker genes *P2RY12* (purinergic receptor P2Y12) and *TREM2* (Supplementary Fig. 2B).

**Fig. 1:**
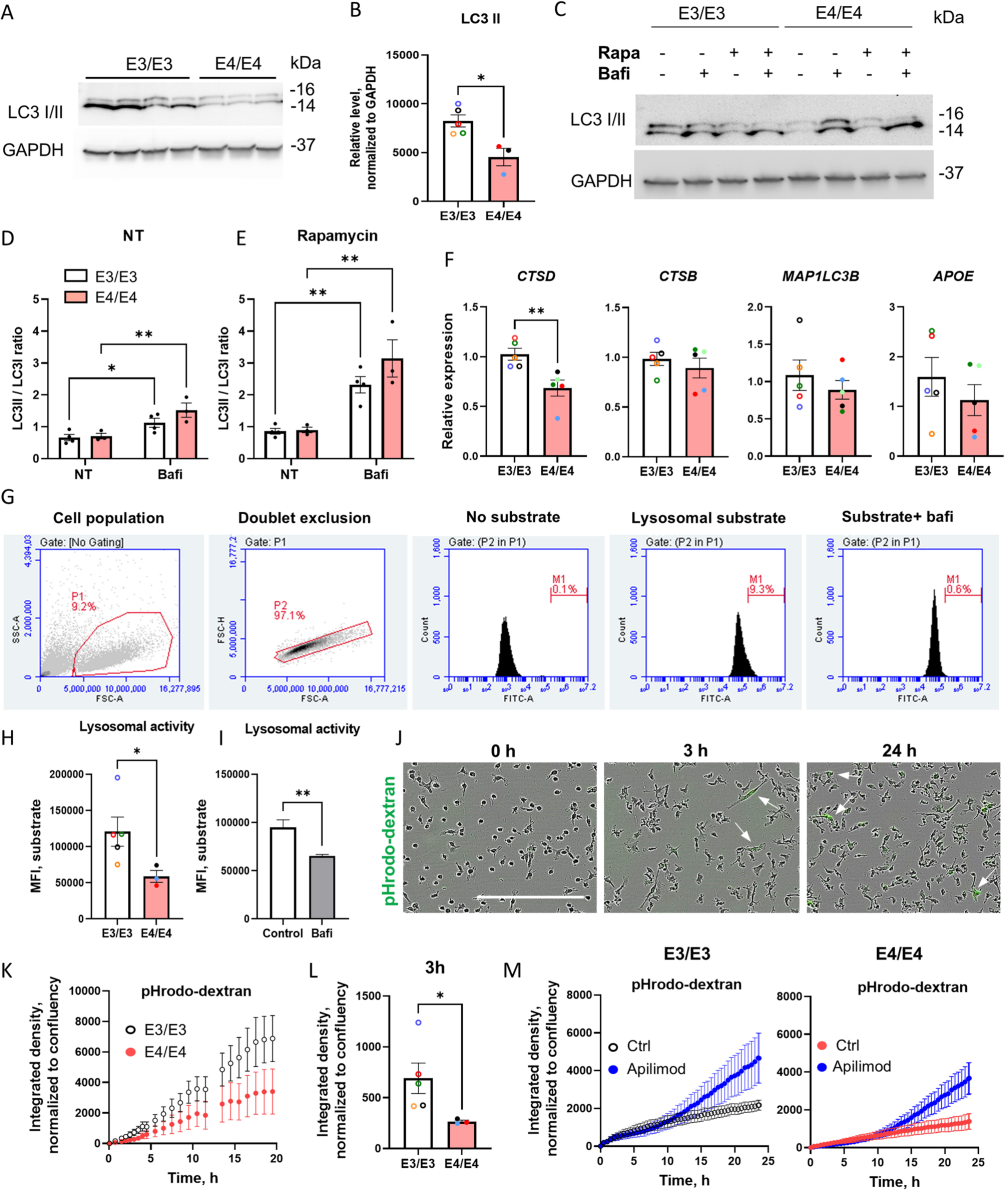
E4/4 iMGs exhibited a decrease in basal levels of LC3-II and macropinocytosis. **A**, representative Western blot images of iMGs in basal conditions. **B**, quantification of the lipidated form of LC3 (II) in iMGs in basal conditions. **C**, representative WB images of iMGs with (+) or without (-) rapamycin (rapa) and bafilomycin (bafi) treatment. **D**, **E**, quantifications of the LC3 (II) to LC3 (I) ratios in iMGs with (+) or without (-) rapamycin (rapa) and bafilomycin (bafi) treatment. F, the relative expression of *CTSD*, *CTSB*, *MAP1LC3B*, and *APOE* mRNA as detected by RT-qPCR. G, the flow cytometry gating strategy, cleaved lysosomal substrate emitted fluorescence in FITC channel. FSC-A, forward scatter area; SSC-A, side scatter area; FSC-H, forward scatter height. H, the mean fluorescence intensity (MFI) of iMGs incubated for 1 h with the lysosomal substrate, which emits fluorescence following lysosomal degradation. I, the MFI of control (E3/E3) iMGs with or without bafilomycin treatment. **J**, **K**, representative images (**J**) and the quantification of pHrodo signal (**K**) in iMGs incubated with pHrodo dextran for different periods of time. Scale bar 300 μm. Arrows, iMGs with dextran in endo-lysosomal compartment. **L**, the quantification of dextran uptake at 3 h. **M**, The quantification of dextran uptake in E3/3 and E4/4 iMGs in the presence or absence of apilimod, *N* = 3 iPSC lines. Individual dots in the bar plots represent the mean values for individual iPSC lines derived from 1–3 independent experiments. The color coding of iPSC lines is shown in Supplementary Table 1. Data in the graphs are shown as mean ± SEM. P values are derived from unpaired t-test (**B**, **F**, **I**), two-way ANOVA (**D**, **E**) or Mann-Whitney test (**H**, **L**). *, *p* < 0.05; **, *p* < 0.01

Autophagy is commonly studied by analyzing the microtubule-associated protein light chain 3 (LC3), which is conjugated to phosphatidylethanolamine (LC3-II), using WB. During autophagy, cytosolic LC3-I is converted into lipidated LC3-II, which associates with nascent phagophore membranes that enclose intracellular material to form autophagosomes. Under basal conditions, E4/E4 iMGs exhibited significantly lower LC3-II levels compared to E3/E3 cells (Fig. 1A, B). As expected, blocking autophagic flux by inhibiting lysosomal acidification with bafilomycin A led to increased LC3-II: LC3-I ratio compared to the basal levels (Fig. 1C, D), but there was no significant difference between the genotypes. Similarly, treatment with rapamycin, an inhibitor of the mTORC1 complex and a well-known autophagy inducer, did not reveal any significant differences between the genotypes (Fig. 1C, E). Mitophagy, where defective mitochondria are degraded by autophagic machinery, is a specific form of autophagy [43]. Mitochondrial mass can be studied using the mitochondrial outer membrane protein TOM-20, which is degraded if mitophagy is increased [44]. Under basal conditions or after treatment with bafilomycin A, there were no differences in TOM-20 protein levels between the genotypes (Supplementary Fig. 2C). Similarly, treatment with rapamycin, with or without bafilomycin A, did not reveal any differences in mitochondrial mass between the genotypes, suggesting similar levels of mitophagy (Supplementary Fig. 2D). Collectively, these results suggest that, compared to E3/E3 iMGs, E4/E4 iMGs exhibit reduced basal levels of LC3-II but no significant differences in basal or rapamycin-induced autophagic flux, indicating that autophagy is not affected by the *APOE* genotype.

Since total levels of LC3 and many other endolysosomal proteins can be transcriptionally regulated by TFEB, the master regulator of lysosomal biosynthesis [45, 46], we assessed the mRNA levels of *MAP1LC3B*, the more abundant of two genes encoding for LC3, and two abundant lysosomal endopeptidases, *CTSD* (cathepsin D) and *CTSB* (cathepsin B). RT-qPCR analysis showed that *CTSD* was significantly downregulated in E4/E4 iMGs, while there was no difference in *CTSB* or *MAP1LC3B* expression between the genotypes (Fig. 1F), suggesting that LC3 protein levels were not primarily regulated by transcription. There was a high variation in the average *APOE* expression between different iPSC lines without a significant genotype effect (Fig. 1F).

Since cathepsins play an essential role in lysosomal degradation, we further analyzed lysosomal activity in iMGs using flow cytometry with an Abcam’s proprietary self-quenched substrate and found that E4/E4 iMGs exhibited significantly lower lysosomal degradation activity compared to E3/E3 cells (Fig. 1G-H). Bafilomycin-treated cells served as a negative control (Fig. 1I). Lysosomal degradation activity can be influenced by the efficiency of substrate uptake into endosomes. Additionally, lipidated LC3-II associates not only with autophagosomes but also with endosomes, macropinosomes, and phagosomes, where it plays a role in vesicle recycling [47, 48]. Therefore, we next investigated endocytosis in iMGs. To assess pinocytosis (fluid-phase endocytosis), we added pHrodo-conjugated dextran (MW 10 kDa) and monitored intracellular fluorescence over 20 h using the IncuCyte live imaging system (Fig. 1J, K). Under basal conditions, pinocytosis was significantly reduced in E4/E4 iMGs compared to E3/E3 iMGs (two-way repeated measures ANOVA, time x genotype *p* = 0.01; Fig. 1J, L). Moreover, when endosome maturation and degradation were blocked by apilimod, a significant accumulation of dextran was observed over 24 h, irrespective of the *APOE* genotype (repeated measures ANOVA, time x treatment *p* < 0.001, Fig. 1M). Interestingly, the *APOE* genotype did not significantly affect the uptake of pHrodo-conjugated zymosan-coated beads (Supplementary Fig. 2E-G) or fibrillar Aβ42 (Supplementary Fig. 2H, I). There was no significant genotype effect on cell migration as evaluated using a Transwell assay, either (Supplementary Fig. 2J). Collectively, these findings indicate that E4/E4 iMGs exhibit reduced endocytosis under basal conditions but no alteration in the rate of phagocytosis.

### APOE 4/4 microglia exhibit reduced metabolic flexibility and dysregulated amino acid metabolism

**Fig. 2:**
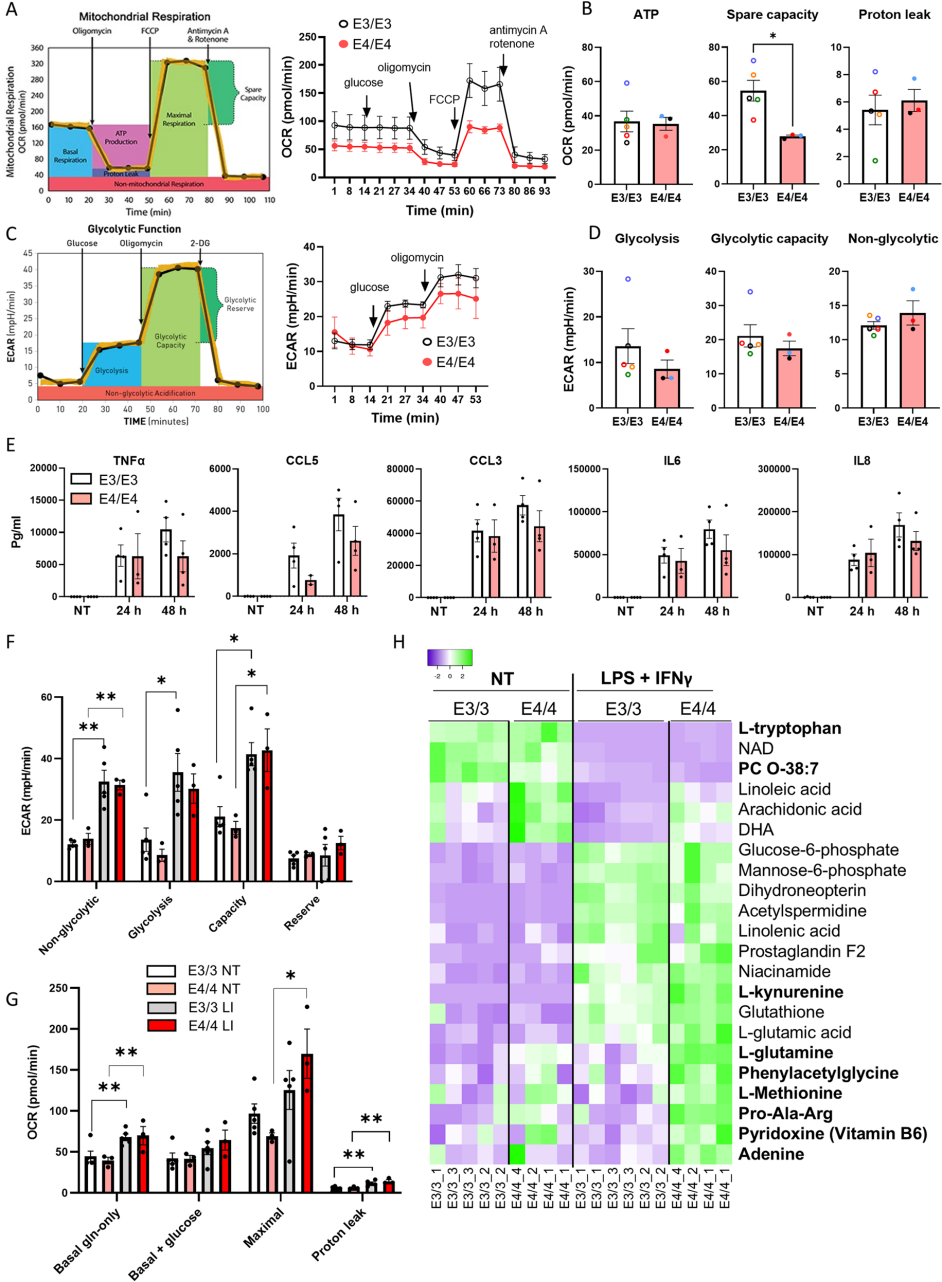
E4/4 iMGs exhibit reduced metabolic flexibility and dysregulated amino acid metabolism. **A**, **C**, the oxygen consumption rate (OCR, **A**) and acidification rate (ECAR, **C**) assessed using the Seahorse analyzer. The graphs show the average of 3–4 iPSC lines obtained from one differentiation batch. **B**, **D**, the quantification of basal ATP production, maximal mitochondrial respiration, proton leak, basal glycolysis, maximal glycolytic capacity, and non-glycolytic acidification. **E**, the levels of cytokines/chemokines released into cell culture medium from iMGs in basal conditions and over 24 h and 48 h of LPS + IFNγ stimulation measured using the Cytometric Bead Array. **F**, non-glycolytic acidification, glycolysis, maximal glycolytic capacity, and glycolytic reserve in iMGs in basal conditions (NT) and following the 24 h of LPS + IFNγ stimulation (LI) as measured using Seahorse analyzer. **G**, the oxygen consumption rate (OCR) in basal conditions in glutamine (gln)-only medium and with the addition of pyruvate and glucose, the maximal and spare respiratory capacity, and protein leak in iMGs in basal conditions (NT) and following the 24 h of LPS + IFNγ stimulation (LI). Individual dots in the bar plots represent the mean values for individual iPSC lines derived from 1–3 independent experiments. The color coding of iPSC lines is shown in Supplementary Table 1. Data in the graphs are shown as mean ± SEM. P values are derived from Mann-Whitney test (**B**) two-way ANOVA with Sidak’s post hoc tests (**F**,**G**). *, *p* < 0.05; **, *p* < 0.01. H, a heatmap of some metabolites up (green)-or downregulated (purple) in iMGs following 48 h of LPS + IFNγ stimulation. The metabolites marked in bold letters were significantly affected by E4/4 genotype. The iPSC lines are indicated at the bottom of the heatmap

Pinocytosis serves as a mechanism to scavenge nutrients and promote cell growth in low-nutrient conditions [49]. The mitochondrial stress test, performed using Agilent Seahorse XFe96 analyzer, revealed that E4/E4 iMGs have similar basal respiration and ATP production but lower maximal respiration capacity compared to E3/E3 cells (Fig. 2A, B). The finding aligns with previous studies suggesting that E4/E4 iMGs may have a reduced ability to rapidly enhance mitochondrial activity in response to an acute increase in energy demand [28]. No significant differences between the genotypes were observed in glycolysis or glycolytic capacity (Fig. 2C, D).

To investigate iMG metabolism under metabolic stress, we induced strong classical activation of iMGs using a combination of bacterial lipopolysaccharide (LPS) and interferon-gamma (IFNγ) (LPS/IFNγ) [50]. As expected, this inflammatory stimulation triggered the secretion of proinflammatory cytokines and chemokines, including TNFα, CCL5, CCL3, IL6, and IL8 (Fig. 2E). Inflammatory stimulation did not significantly affect iMG cell confluency, with no notable differences between genotypes (Supplementary Fig. 3E). To meet the increased energy demand during inflammation, microglia must significantly enhance their metabolic rate. Accordingly, LPS/IFNγ stimulation induced glycolysis, glycolytic capacity, basal and maximal respiration, and proton leak in mitochondria, with no significant effect of the *APOE* genotype (Fig. 2F, G).

When iMGs were subjected to 48-h LPS/IFNγ inflammatory stimulation and analyzed using un-targeted metabolomics, principal component analysis (PCA) of normalized data revealed two distinct clusters corresponding to unstimulated and stimulated cells, confirming that inflammatory stimulation induced metabolic reprogramming in iMGs (Supplementary Fig. 3A, B). Figure 2H highlights selected metabolites altered by LPS/IFNγ treatment, including amino acids, fatty acids, and glucose metabolites. While *APOE* genotypes did not cause clear clustering of the samples, a total of 50 molecular features were differentially produced (q-value < 0.1) between the E3/E3 and E4/E4 iMGs in the unstimulated group and 132 molecular features in the stimulated group. Ultimately, nine significantly altered metabolites were reliably identified: L-glutamine and plasmalogen PC O-38:7 under basal conditions (Fig. 2H; Table S1) and seven metabolites (L-tryptophan, L-methionine, L-kynurenine, phenylacetylglycine, tripeptide Pro-Ala-Arg, adenine, and pyridoxine) in the stimulated group. All identified metabolites were elevated in E4/E4 iMGs, except for plasmalogen PC O-38:7 (Fig. 2H). The exact identity of this plasmalogen could not be determined from the data alone; however, in mammalian cells, the most likely candidate is PC(P-16:0/22:6(4Z,7Z,10Z,13Z,16Z,19Z)), with the longer fatty acid being docosahexaenoic acid (DHA) [51]. Since inflammation induces the hydrolysis of plasmalogens to generate pro- or anti-inflammatory mediators [52], a lower basal level of plasmalogen in E4/E4 iMGs may indicate a chronic inflammatory state. There was no significant *APOE* genotype effect on cholesterol levels, and this was confirmed using Amplex Red assay kit in a separate differentiation batch (Supplementary Fig. 3C). Our metabolomics setup did not allow for reliable measurement of non-polar lipids such as triglycerides. Collectively, our metabolomics results demonstrate that E4/4 iMGs exhibit dysregulated amino acid and phospholipid metabolism.

### Inflammation induces lysosomal dysfunction in iMG

To verify that LPS/IFNγ treatment changes the levels of key proteins regulating amino acid metabolism, we conducted proteomic analysis of five control E3/E3 iMG lines (Supplementary Fig. 3D). In line with the metabolomics data and existing literature [53], we detected an upregulation in the levels of tryptophan-metabolizing proteins (indoleamine 2,3-dioxygenase (IDO)1, kynurenine 3-monooxygenase (KMO) and kynureninase (KYNU)) following the 48 h LPS/IFNγ stimulation (Table S2). We also observed increased levels of some proteins involved in glutamine uptake (SLC1A5), synthesis (GLUL) and hydrolysis (ASNS) (Table S2). When comparing the proteome of two (isogenic to each other) E4/E4 lines with E3/E3 lines, no significant differences (threshold 2-fold change, *p* < 0.05) were observed in the levels of the proteins involved in the tryptophan or glutamine metabolism (Tables S4, S5).

**Fig. 3:**
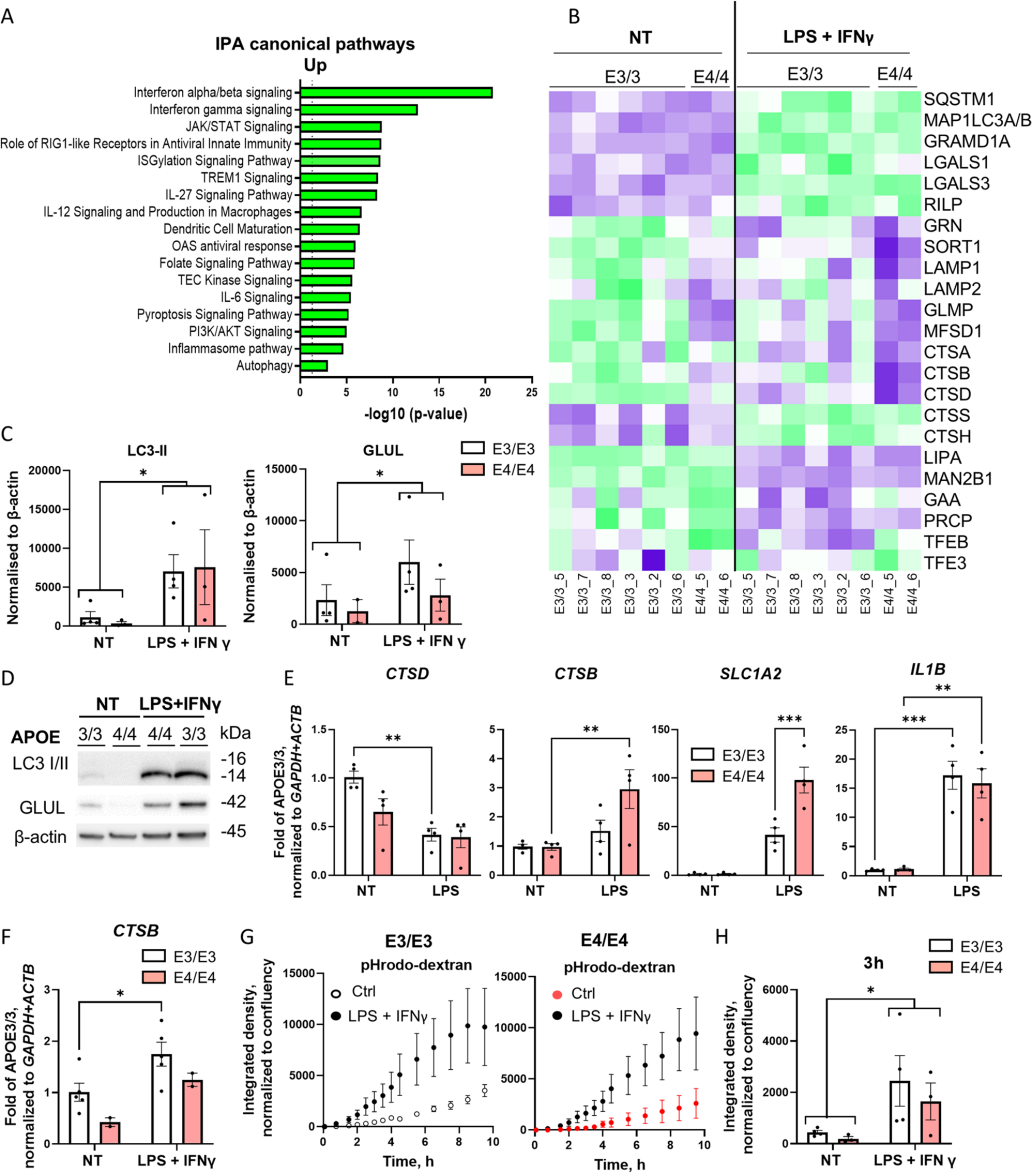
Inflammation induces lysosomal dysfunction in iMGs. **A**, top enriched pathways for proteins upregulated in LPS + IFNγ-stimulated E3E/3 iMGs identified using Ingenuity Pathway Analysis. **B**, the heatmap of selected lysosomal proteins in iMGs in basal conditions (NT) and after 48 h of LPS + IFNγ stimulation. **C**, **D** the representative WB images and the quantification analysis of LC3II and GLUL in iMGs in basal conditions (NT) and after 48 h of LPS + IFNγ stimulation. **E**, the relative expression of *CTSD*, *CTSB*, *SLC1A2*, and *IL1B* mRNA in basal conditions (NT) and after 24 h of LPS stimulation as detected by RT-qPCR. **F**, the relative expression of *CTSB* in basal conditions (NT) and after 48 h of LPS/IFNγ stimulation as detected by RT-qPCR. G, H, the quantification of pHrodo dextran uptake in E3/E3 and E4/E4 iMGs in basal conditions (NT) and after 24 h LPS + IFNγ treatment. Data in the graphs are shown as mean ± SEM. Individual dots in the bar plots represent the mean values for individual iPSC lines derived from 1–3 independent experiments. P values are derived from two-way ANOVA with Sidak’s posthoc test; *, *p* < 0.05; **, *p* < 0.01; ***, *p* < 0.001

The IPA analysis showed that in addition to common inflammatory pathways, such as interferon alpha/beta signaling, interferon gamma signaling and inflammasome pathway, LPS/IFNγ treatment induced PI3K/AKT signaling and autophagy pathways (Fig. 3A). The autophagy pathway included LC3 (MAP1LC3A/B) and SQSTM1/p62, an autophagosomal cargo protein targeting ubiquitinated substrates for degradation and linking autophagy to the activation of pro-inflammatory transcription factor nuclear factor kappa B (NF-κB) [54] (Fig. 3B, Table S3). We also observed increased levels of cholesterol transfer protein GRAMD1A (Fig. 3B, Table S2), which was recently identified as a regulator of autophagosome biogenesis [55]. In contrast, inflammatory stimulation decreased the levels of several lysosomal proteases, including cathepsin D (CTSD), lysosomal acid lipase (LIPA), lysosomal alpha-mannosidase (MAN2B1), and lysosomal pro-X carboxypeptidase (PRCP) (Fig. 3B, Table S2), suggesting that strong inflammatory activation may impair lysosomal degradation.

To validate the alterations identified by proteomic analysis, immunoblotting was done using the samples from an independent experiment. Consistent with the proteomics data, this WB analysis confirmed that 48-h stimulation with LPS/IFNγ strongly increased the accumulation of LC3-II as well as glutamine synthetase (GLUL), although the protein levels were not affected by the *APOE* genotype (Fig. 3C, D).

To investigate whether the differences in protein levels were determined by transcriptional regulation, we performed RT-qPCR analysis on iMGs treated with LPS for 24 h. In agreement with the proteomics data, *CTSD* mRNA expression was significantly reduced following the LPS treatment (Fig. 3E). In contrast, *CTSB* mRNA was significantly upregulated by both LPS alone (two-way ANOVA, *p* = 0.015; Fig. 3E) and LPS/IFNγ stimulation (two-way ANOVA, *p* = 0.015; Fig. 3F), which in combination with lower protein levels at 48 h suggested increased degradation or leakage of cathepsin B. The glutamate transporter SLC1A2 has recently been implicated in inflammatory responses in macrophages by sustaining macropinocytosis and mTORC1 activation [56]. While our iMGs did not express *SLC1A2* under basal condition, the expression was upregulated in response to LPS stimulation and was significantly stronger in E4/E4 iMGs compared to E3/E3 iMGs (Fig. 3E). In contrast, there was no *APOE* genotype effect on the mRNA levels of *IL1B* (Fig. 3E). LPS/IFNγ stimulation increased the rate of pinocytosis in both E3/E3 and E4/E4 iMGs (Fig. 3G, H), thereby placing a greater burden on the endo-lysosomal system.

### APOE 4/4 iMGs exhibit higher lysosomal membrane permeabilization upon Aβ42 exposure

**Fig. 4:**
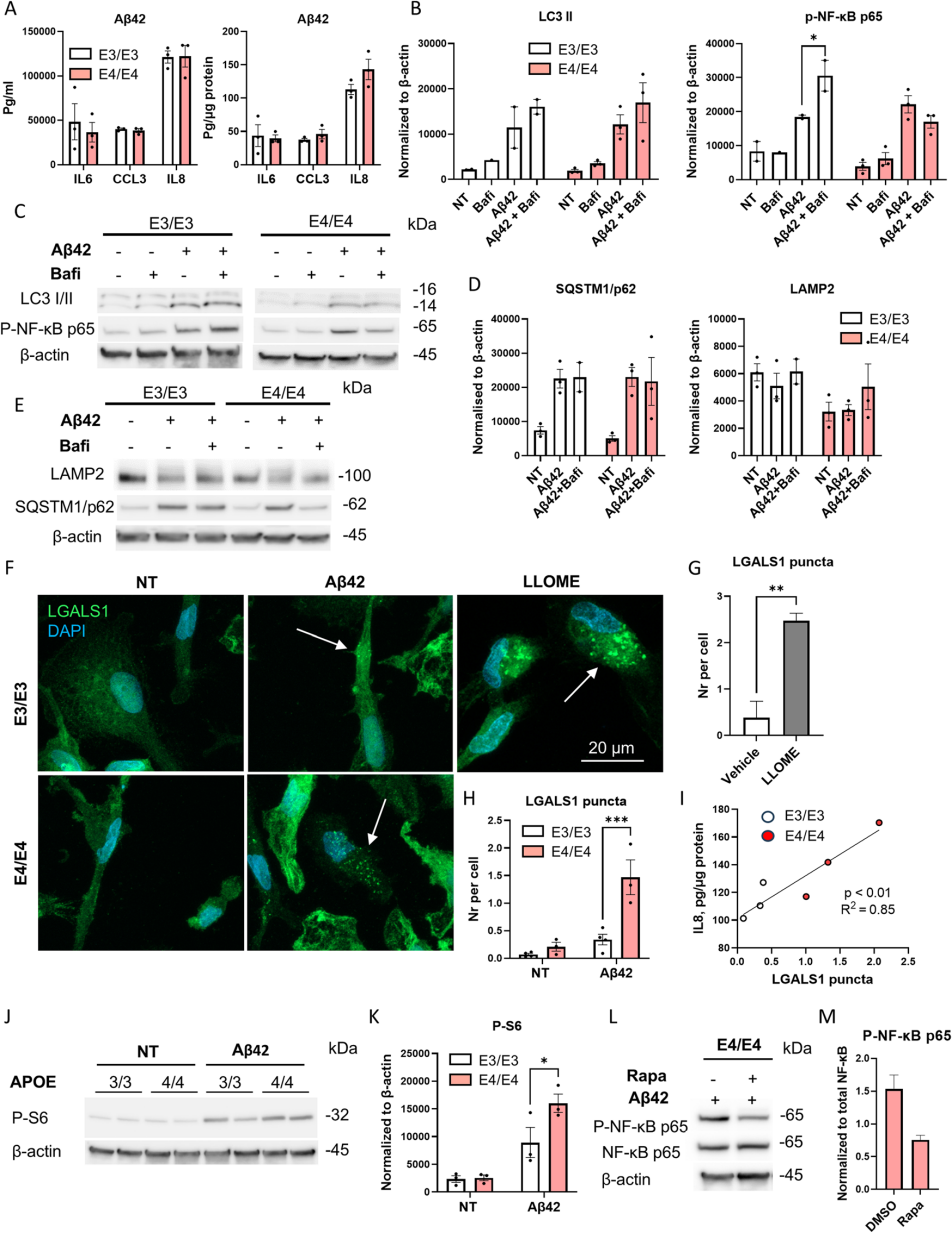
E4/E4 iMGs exhibit higher lysosomal membrane permeabilization upon Aβ42 exposure. **A**, the levels of cytokines/chemokines released into cell culture medium from iMGs over 48 h of Aβ42 stimulation measured using the Cytometric Bead Array not normalized (pg/ml) and normalized to the protein content in cell lysates (pg/µg protein). **B**-**E**, the representative WB images (**C**, **E**) and quantification data (**B**, **D**) of iMGs treated with (+) and without (-) Aβ42 and bafilomycin (bafi) for 48 h. F-H, representative images of LGALS1 staining (**F**) and quantification data of LGALS1 puncta (**G**, **H**) in iMGs treated with LLOME, Aβ42, or left untreated (NT). Scale bar 20 μm. Arrows, iMGs with LGALS1 puncta. **I**, Correlation of the levels of secreted IL8 (y-axis) and LGALS1 puncta (x-axis) in iMGs after Aβ42 stimulation. P value and R squared value derived from Simple linear regression analysis. J-M the quantification (**K**, **M**) and representative WB images (**J**, **L**) of phospho-S6 ribosomal protein (Ser235/236) and phospho-NF-κB p65 (Ser536). Data in the graphs are shown as mean ± SEM. Individual dots in the bar plots represent the mean values for individual iPSC lines. P values are derived from two-way repeated measures ANOVA with Sidak’s posthoc tests (**B**, **D**, **H**, **K**) or unpaired t-test (**G**). *, *p* < 0.05; **, *p* < 0.01; ***, *p* < 0.001

To determine whether comparable results could be achieved using an alternative inflammatory stimulus, more relevant for AD, we treated iMGs for 48 h with soluble Aβ42 oligomers containing 0.1 EU endotoxin per µg peptide. First, we measured cytokine secretion after Aβ42 treatment using CBA as above and found that iMGs of both genotypes secreted cytokines at levels comparable to those observed after LPS/IFNγ treatment (Fig. 4A).

Further, we analyzed LC3-II levels by WB, and found that, similarly to LPS/IFNγ stimulation, Aβ42 strongly increased intracellular LC3-II (*p* = 0.04), irrespective of the *APOE* genotype (Fig. 4B, C). Bafilomycin treatment did not further increase LC3-II levels, suggesting that Aβ42 stimulation blocked lysosomal degradation. Similarly to LC3-II, SQSTM1/p62, levels were significantly increased following Aβ42 stimulation (*p* = 0.001); however, adding bafilomycin to Aβ42-stimulated iMGs did not result in a significant change (Fig. 4D, E). Interestingly, lysosomal-associated membrane protein 2 (LAMP-2), a key mediator of autophagosome-lysosome fusion [57, 58], was unaffected by Aβ42 stimulation but tended to be reduced in E4/E4 iMGs (Fig. 4D, E). Since proteomic analysis comparing two (isogenic to each other) E4/E4 lines to E3/E3 lines revealed a downregulation of two other lysosomal proteins MFSD1 and GLMP in E4/E4 iMGs at basal conditions (Fig. 3B, Table S4), the overall lysosomal protein content may be reduced by E4/E4 genotype.

Phospho-NF-κB p65 (Ser536) is an active form of NF-κB (subunit p65), a key mediator of inflammation. As expected, Aβ42 stimulation significantly increased p-NF-κB p65 levels (*p* = 0.02; Fig. 4B, C). Interestingly, inhibition of lysosomal acidification with bafilomycin further increased p-NF-κB p65 in E3/E3 iMGs but not in E4/E4 iMGs, suggesting that lysosomal enzyme activity selectively affected the inflammatory response in E3/E3 iMGs.

Since the accumulation of SQSTM1/p62 and LC3-II following Aβ42 exposure suggested impaired lysosomal degradation and elevated lysosomal stress, we next analyzed lysosomal membrane permeabilization (LMP) using the lysosomal galectin puncta assay [59]. As shown in Fig. 4F, H, the formation of galectin puncta, detected with galectin (LGALS)-1-specific antibody, was significantly higher in E4/E4 iMGs than in E3/E3 iMGs after Aβ42 exposure, indicating increased lysosomal leakage in E4/E4 iMGs. A two-hour treatment with LLOME served as a positive control (Fig. 4F, G). Interestingly, the levels of secreted IL8 significantly correlated with galectin puncta after Aβ42 stimulation, suggesting that lysosomal leakage exacerbated the inflammatory response (Fig. 4I). It has been demonstrated that IL8 production can be regulated by mTORC1 activity [60]. Since mTORC1 is a critical regulator of lysosomal biogenesis and autophagy [45, 61], we assessed its activity via WB by measuring phosphorylation of the S6 ribosomal protein (Ser235/236), a downstream component of mTORC1 signaling complex. Figures 4J, K show that phosphorylated S6 levels were significantly higher in E4/E4 iMGs stimulated with Aβ42 as compared to E3/E3 iMGs, indicating increased mTORC1 activity in E4/E4 cells. Since mTORC1 can promote microglial proliferation [62], we performed KI67 immunostaining to visualize proliferating cells. Aβ42 stimulation significantly increased cell proliferation; however, no effect of the *APOE* genotype was observed under either basal or Aβ42-stimulated conditions (Supplementary Fig. 4C, D). As expected, treatment with the mTORC1 inhibitor rapamycin reduced NF-κB p65 phosphorylation [63] by half in an E4/E4 line (Fig. 4L, M), suggesting that mTORC1 activity promoted inflammatory response in E4/E4 iMGs. In contrast to a recent study by Haney and coworkers [64], we did not find lipid droplet accumulation following Aβ42 stimulation and there were no differences between the genotypes (Supplementary Fig. 4A, B).

## Discussion

The ε4 isoform of ApoE is the most prevalent genetic risk factor for AD. In this study, E4/E4 iMGs showed diminished basal levels of pinocytosis and lipidated LC3 in vitro. In macrophages and microglia, constitutive pinocytosis primarily functions to continuously sample the environment for pathogens [65]. Additionally, pinocytosis has been shown to facilitate the uptake and clearance of soluble Aβ species [66]. In agreement with our study, Lin and coworkers have previously reported reduced uptake of fluorescent soluble Aβ42 by E4/E4 iMGs [15]. Thus, E4/E4 microglia may be less responsive to homeostatic perturbations and less efficient at clearing soluble waste, potentially contributing to impaired proteostasis in AD. Endocytosis defects have also been previously reported in E4/E4 astrocytes [67]. Contrary to the findings of Haney and coworkers [64], we did not observe significant differences in the uptake of solid particles, such as zymosan-coated beads or fibrillar Aβ42. However, Haney and coworkers used only one isogenic pair of iPSC lines to conduct the experiment. In our study, E4/E4 iMGs exhibited lower level of pHrodo zymosan-coated bead phagocytosis than isogenic E3/E3 cells (Supplementary Fig. 2D red open circle vs. red circle). Thus, other genetic factors likely contributed to the high within-group variation in the uptake of zymosan beads, preventing us from observing a significant *APOE* genotype effect. Interestingly, Konttinen et al. [28] reported a slight reduction in the numbers of internalized zymosan beads in E4/E4 iMGs, but did not observe any difference in fluorescence intensity. Overall, these results suggest that the *APOE* genotype has a stronger impact on fluid-phase endocytosis than on phagocytosis.

In our study, inflammatory stimulation strongly increased autophagosome formation, pinocytosis, and mTORC1 activity, while blocking autophagic flux and abolishing genotype-related differences. The blockade of autophagic flux was confirmed by elevated SQSTM1/p62 levels, indicating that inflammatory conditions override basal autophagic and endocytic differences between *APOE* genotypes.

Both autophagic and endocytic pathways culminate in lysosomal degradation. Substrate overload can lead to LMP, allowing intralysosomal components such as cathepsins to be released into the cytoplasm, further impairing lysosomal protein degradation [68]. LMP, a hallmark of lysosomal dysfunction, has been implicated in neurodegeneration and chronic inflammation. Interestingly, we observed an increased number of LGALS1-positive puncta in Aβ42-stimulated E4/E4 iMGs, suggesting elevated LMP. Since damaged lysosomes are cleared by autophagy (lysophagy) [69], an increased number of LGALS1 puncta may also indicate impaired lysophagy. LMP can further induce inflammasome activation in macrophages and microglia [70–73], a process which can be triggered by the leakage of active cathepsin B from lysosomes into the cytosol [71, 72]. Overall, these findings suggest that E4/E4 microglia exhibit heightened vulnerability to lysosomal leakage and associated inflammatory cascades, potentially contributing to chronic neuroinflammation in AD. Although we did not directly assess inflammasome activation or cathepsin B activity in our study, we found a significant correlation between LMP and secreted IL8 (CXCL8), a major neutrophil chemokine known to be involved in AD [74] and regulated by inflammasome activation [75]. Furthermore, we found that bafilomycin, an inhibitor of lysosomal acidification and protease activity, exhibited a proinflammatory effect in E3/E3 iMGs, but not in E4/E4 iMGs, indicating genotype-dependent differences in lysosomal regulation of inflammation. In human iPSC-derived macrophages, LMP has recently been linked to metabolic reprogramming in mitochondria [76], and the LMP-induced changes in mitochondrial respiration and glycolysis were similar to those we observed in iMGs following LPS/IFNγ stimulation. The mTORC1 pathway is known to be the master regulator of metabolic reprogramming stimulating protein synthesis, promoting aerobic glycolysis, suppressing autophagy, and reducing lysosomal acidification [45, 61, 77–79]. In our study, mTORC1 was significantly stronger activated in E4/E4 iMGs following Aβ42 stimulation. This finding indicates that targeting mTORC1 signaling could be beneficial in mitigating lysosomal dysfunction and inflammation in ApoE ε4-associated neurodegeneration.

Our results are consistent with previous studies in murine models, where prolonged exposure to Aβ42 resulted in the accumulation of autophagosomes, LMP, and cathepsin D leakage in murine microglial cell line [80]. Although it has been shown before that E4/E4 potentiates LMP in neuroblastoma cells after Aβ treatment in vitro [81, 82], our study is the first to demonstrate the effect of E4/E4 genotype on LMP in human microglia, which play a central role in late-onset AD pathophysiology.

Some earlier studies associated lysosomal dysfunction with cholesterol accumulation inside lysosomes in E4/E4 glia, particularly astrocytes [16, 19]. We did not detect *APOE* genotype effect on intracellular cholesterol levels or lipid droplets. However, we cannot exclude the possibility that the presence of ApoE ε4 affected the distribution of cholesterol between different cellular compartments. Also, our metabolomics setup did not allow for the reliable measurement of cellular triglycerides.

Interestingly, we observed elevated levels of amino acids, particularly L-glutamine, in E4/E4 iMGs. This was accompanied by increased mRNA expression of the glutamate transporter *SLC1A2* following inflammatory stimulation. Increased levels of L-glutamine were also reported in primary microglia from E4/E4 targeted replacement mice [83]. Glutamine and glutamate metabolism are tightly linked. Glutaminase (GLS) catalyzes glutamine conversion into glutamate, thus facilitating its utilization in the TCA cycle and activating mTORC1 [79]. In microglia/macrophages, M1 polarization and inflammasome activation require increased glutamine utilization in the TCA cycle [79, 84]. Our mitochondrial stress test results suggested that LPS/IFNγ stimulation increased glutamine utilization irrespective of the *APOE* genotype. However, due to higher *SLC1A2* mRNA expression it is possible that instead of enhanced glutamine-to-glutamate conversion, E4/E4 iMGs exhibit increased lysosomal glutamate efflux, sustaining mTORC1 activation [56]. Additionally, the elevated kynurenine levels detected in E4/E4 iMGs after LPS/IFNγ stimulation may have contributed to mTORC1 activation, as kynurenine has been shown to directly activate mTORC1 in human lymphocytes [85].

Although we did not detect *APOE* genotype-associated differences in basal autophagic flux, we found that E4/E4 iMGs exhibited lower expression level of key lysosomal markers, including cathepsin D and LAMP2, suggesting a reduced lysosomal content. These results are consistent with the findings by TCW and coworkers [16] reporting downregulation in lysosomal genes in E4/E4 iMGs. Notably, LAMP2 overexpression in ischemic cardiomyocytes has been shown to restore autophagic flux, promote cathepsin trafficking, and mitigate LMP [86]. These results highlight the potential therapeutic relevance of enhancing lysosomal biogenesis in *APOE*4/4 carriers.

We could not replicate previously reported glycolytic abnormalities in E4/E4 iMGs. Prior studies have reported conflicting data regarding aerobic glycolysis levels in E4/E4 iMGs. While Konttinen et al. [28] reported a significant decrease in glycolysis and glycolytic capacity, Victor et al. [29] found upregulation of the glucose transporters GLUT1 (SLC2A1) and GLUT3, suggesting increased glycolysis. Additionally, a recent study of mouse E4/E4 microglia reported elevated aerobic glycolysis levels [83]. Given that glycolysis is often linked to inflammation-driven metabolic reprogramming, its levels may be highly dependent on experimental conditions.

## Conclusion

Our study provides new insights into ApoE ε4-mediated alterations in microglial lysosomal function, metabolism, and inflammation. Our findings suggest that inflammatory stimulation causes mTORC1-mediated metabolic reprogramming in human iPSC-derived microglia, which leads to lysosomal stress and the suppression of autophagic flux. These effects were exacerbated in Aβ42-stimulated microglia homozygous for *APOE* ε4 allele. Given the role of microglia in AD pathogenesis, these results highlight the potential of targeting lysosomal function and mTORC1 signaling as therapeutic strategies for ApoE ε4-associated neurodegeneration. Since this study used only simple monocultures, the results need to be validated in more complex models that include other brain cell types, such as neurons and astrocytes.

## Electronic supplementary material

Below is the link to the electronic supplementary material.

Supplementary Material 1

Supplementary Material 2

Supplementary Material 3

## Data Availability

No datasets were generated or analysed during the current study.

## Abbreviations

AD: Alzheimer’s disease
ApoE: Apolipoprotein E
Aβ: Beta-amyloid
Bafi: Bafilomycin
CBA: Cytometric bead array
CCL3: C-C Motif chemokine ligand 3, MIP1α
CCL5: C-C Motif chemokine ligand 5, RANTES
CLEAR: Coordinated lysosomal expression and regulation
CTSB: Cathepsin B
CTSD: Cathepsin D
CX3CL1: C-X3-C motif chemokine ligand 1
DMSO: Dimethyl sulfoxide
D-PBS: Dulbecco’s phosphate-buffered saline
E3: ApoE isoform ε3
E4: ApoE isoform ε4
ECAR: Extracellular acidification rate
FCCP: Carbonyl cyanide-4 (trifluoromethoxy) phenylhydrazone
FDR: False discovery rate
GLUL: Glutamine synthetase
HIFP: 1,1,1,3,3,3-Hexafluoro-2-propanol
IFNγ: Interferon-gamma
IL: Interleukin
iMG: Induced pluripotent stem cell-derived microglia
IPA: Ingenuity Pathway Analysis
iPSC: Induced pluripotent stem cell
LAMP-2: Lysosomal-associated membrane protein 2
LC3: Microtubule-associated protein light chain 3
LC3- II: LC3 conjugated to phosphatidylethanolamine
LLOMe: L-leucyl-L-leucine methyl ester
LMP: Lysosomal membrane permeabilization
LPS: Lipopolysaccharide
M-CSF: Macrophage colony-stimulating factor
MFI: Mean fluorescence intensity
MITF: Microphthalmia-associated transcription factor
mTORC: Mammalian target of rapamycin
NF-κB: Nuclear factor kappa B subunit
NT: Basal conditions/untreated
OCR: Oxygen consumption rate
RAPA: Rapamycin
RT: Room temperature
RT-qPCR: Reverse transcription quantitative real-time PCR
SQSTM1/p62: Sequestosome 1
TBST: TBS-0.05% Tween
TFEB: Transcription factor EB
TNFα: Tumor necrosis factor alpha
TREM2: Triggering receptor expressed on myeloid cells 2
WB: Western Blot
