# Supplementary material for: Inflammation-induced lysosomal dysfunction in human iPSC-derived microglia is exacerbated by APOE 4/4 genotype
Source: J Neuroinflammation. 2025 Jun 2;22:147. doi: 10.1186/s12974-025-03470-y (PMC12131611; doi:10.1186/s12974-025-03470-y)
Supplement: Supplementary file 1 — Supplementary Material 1 [file 12974_2025_3470_MOESM1_ESM.docx]

- 1. Supplementary materials

**Table 1.** The summary of the human-derived iPSC lines used in this study.

| **Represent symbol** | **Line** | **Donor’s sex** | **Donor’s**  **age at biopsy** | ***APOE* genotype** | **Health status** | **Reference** |
| --- | --- | --- | --- | --- | --- | --- |
|  |  |  |  |  |  |  |
|  | E3/3_1 | M | 67 | ε3/ ε3 | Healthy | [1] |
|  | E3/3_2 | M | 64 | ε3/ ε3 | Healthy | [2] |
|  | E3/3_3  E3/3_4  E3/3_5 | M  M  M | 63  66  80 | ε3/ ε3  ε3/ ε3  ε3/ ε3 | Healthy  Healthy  Isogenic of E4/4_5 | [2]  [1]  [3] |
|  | E3/3_6  E3/3_7 | M  F | 80  77 | ε3/ ε3  ε3/ ε3 | Isogenic of E4/4_5,  PLCG2 P522R  Healthy | [4]  [5] |
|  | E3/3_8 | F | 77 | ε3/ ε3 | Healthy | [5] |
|  |  |  |  |  |  |  |
|  | E4/4_1 | M | 58 | ε4/ ε4 | Healthy | [1] |
|  | E4/4_2  E4/4_3  E4/4_4 | M  M  M | 61  63  68 | ε4/ ε4  ε4/ ε4  ε4/ ε4 | Healthy  Healthy  NPH/AD* | Sup. Fig.1  Sup. Fig.1  Sup. Fig.1 |
|  | E4/4_5  E4/4_6 | M  M | 80  80 | ε4/ ε4  ε4/ ε4 | AD*  Isogenic of E4/4_5,  PLCG2 P522R | [3, 4]  [4] |

*NPH, Normal pressure hydrocephalus; AD, Alzheimer’s disease

- 1. Immunocytochemistry

The astrocytes used for immunocytochemistry were differentiated according to a protocol by Oksanen et al 2017 [5]. The cells were fixed with 4% paraformaldehyde in phosphate-buffered saline (PBS) at room temperature (RT) for 20 min. The cells were permeabilized with 0.2% Triton X-100 in PBS at RT for 10 min. The unspecific binding sites were blocked with 1% bovine serum albumin in 0.05 % Tween-20 in PBS at RT for 1h. For Ki67 staining, the iMGs were permeabilized and unspecific binding sites blocked with 0.3% Triton X-100 in 5% normal goat serum (NGS) in PBS at RT for 1h. For Tra1-81 and SSEA4 staining, the cells were not permeabilized. The primary and secondary antibodies and dilutions used are shown in Table 2. CD18 primary antibody was a generous gift from Prof. C. G. Gahmberg, University of Helsinki. Nuclei were visualized by DAPI staining (Sigma) at RT. The coverslips were mounted with Fluoromount, and images were acquired with a Zeiss AxioImager M1 epifluorescence microscope or EVOS M5000 (Invitrogen by Thermo Fisher Scientific) at 10x magnification. The percentage of Ki67 positive nuclei were quantified with ImageJ Software.

**Table 2.** Primary and secondary antibodies used in immunohistochemistry and immunocytochemistry.

| **Primary antibody** | **Host** | **Specificity** | **Supplier** | **Catalog #** | **Application** | **Dilution** | **RRID** |
| --- | --- | --- | --- | --- | --- | --- | --- |
| Iba1 | goat | human | Novus | NB100-1028 | ICC | 1:200 | AB_521594 |
| CX3CR1 | rabbit | human | Thermo Fisher | PA5-19910 | ICC | 1:100 | AB_11154568 |
| TREM2 | goat | human | R&D Systems | AF1828 | ICC | 1:40 | AB_2208689 |
| Iba1 | rabbit | human, mouse | Wako | 019-19741 | ICC | 1:500 | AB_839504 |
| Ki67 | rat | mouse, non-human primate, rat, cynomolgus monkey, human, dog | eBioscience | 14-5698-82 | ICC | 1:200 | AB_10854564 |
| LGALS1 | rabbit | human | Abcam | ab25138 | ICC | 1:1000 | AB_2136615 |
| Phospho-NF-κB p65 (Ser536) | rabbit | human, mouse, rat, hamster, monkey, pig | Cell Signalling | 3033 | WB | 1:1000 | AB_331284 |
| Total- NF-κB p65 (D14E12) XP | rabbit | human, mouse, rat, hamster, monkey, dog | Cell Signalling | 8242 | WB | 1:1000 | AB_10859369 |
| Phospho-S6 Ribosomal protein (Ser235/236) | rabbit | human, mouse, rat | Cell Signalling | 4857 | WB | 1:1000 | AB_2181035 |
| LC3 A/B | rabbit | human, mouse, rat | Cell Signalling | 4108 | WB | 1:1000 | AB_2137703 |
| GLUL | rabbit | human, mouse | Abcam | ab197024 | WB | 1:5000 |  |
| LAMP2 | rabbit | human, mouse, rat | Sigma-Aldrich | L0668 | WB | 1:1000 | AB_477154 |
| SQSTM1/p62 | mouse | human | Abcam | ab56416 | WB | 1:1000 | AB_945626 |
| OCT4 | mouse | human | Merck Millipore | MAB4401 | ICC | 1:400 |  |
| NANOG | goat | human | R&D Systems | AF1997 | ICC | 1:100 | AB_355097 |
| TRA1–81 | mouse | human | Merck Millipore | MAB4381 | ICC | 1:200 | AB_177638 |
| SSEA4 | mouse | human | Merck Millipore | MAB4304 | ICC | 1:400 | AB_177629 |
| TOM-20 (F-10) | mouse | human | Santa Cruz Biotechnology | sc-17764 | WB | 1:1000 | AB_628381 |
| β-actin | mouse | human, mouse, rat, pig, bovine, canine, feline, rabbit, carp, guinea pig, chicken, sheep | Sigma-Aldrich | A5441 | WB | 1:10000 | AB_476744 |
| GAPDH | rabbit | human, mouse, rat | Cell Signalling | 2118 | WB | 1:1000 | AB_561053 |
| **Secondary antibodies** |  |  |  |  |  |  |  |
| Alexa Fluor 488 | chicken | goat | Thermo  Fisher | A21467 | ICC | 1:300 | AB_2535870 |
| Alexa Fluor 488 | goat | mouse | Thermo Fisher | A11001 | ICC | 1:300 | AB_2534069 |
| Alexa Fluor 568 | goat | rabbit | Thermo Fisher | A11011 | ICC | 1:300 | AB_143157 |
| Alexa Fluor 568 | goat | rat | Thermo Fisher | A11077 | ICC | 1:300 | AB_2534121 |
| anti-mouse HRP | rabbit | mouse | Sigma-Aldrich | A9044 | WB | 1:20000 | AB_258431 |
| anti-rabbit HRP | goat | rabbit | Invitrogen | A16096 | WB | 1:20000 | AB_2534770 |

- 1. Phagocytosis assays

For pHrodo zymosan phagocytosis assays, the cells were plated at a density of 20,000 cells per well into a black-walled 96-well imaging plate in 90 μl iMG medium containing the maturation factors CD200 and CX3CL1 three days before performing the assay. A day before the assay, 50 μl of fresh maturation iMG medium was added per well. On the day of the phagocytosis assay 40 μl of the medium was removed to keep the total volume at 100 μl. pHrodoTM zymosan BioParticlesTM were freshly dissolved in PBS, sonicated according to the manufacturer’s instructions, and added on top of cells at a final concentration of 83.3 μg/ml. The phagocytosis assay was performed using the IncuCyte S3 live cell imaging system (Sartorius) at the Biomedicum Stem Cell Center core facility, University of Helsinki. The cells were imaged every 30 minutes for 6.5 h and the integrated intensity of the fluorescence signal was normalized to cell confluence assessed by IncuCyte S3 before adding the pHrodo zymosan BioParticles.

To investigate fibrillar Aβ42 uptake by microglia, cells were re-plated at 222,000 cells per well in 1 ml iMG medium on a Matrigel-coated 12-well plate. Maturation factors were added to the medium at 4 days prior to the assay. Human beta-amyloid (1-42) HiLyte^TM^ Fluor 488-labeled (AnaSpec) was reconstituted in PBS at 0.1 mg/ml and fibrillized for five days at + 37 °C in a glass vial. On the day of the assay, Aβ42 HiLyteTM Fluor 488-labeled fibrils were sonicated and incubated for 2 h on top of cells at the final concentration of 5 μg/ml at 37 °C. Then the cells were washed with ice-cold PBS and harvested by scraping and centrifuging at 300 G 5 min +4 °C. The cells were stained for 45 min with anti-CD11b APC-conjugated antibody (Biolegend) in 1% BSA. The unspecific binding was blocked by the human Fc receptor binding inhibitor (Thermo Fisher Scientific). The cells were analyzed using BD Accuri C6 flow cytometer with BD CSampler Plus software (BD Biosciences, Franklin Lakes, NJ, USA) at the Biomedicum Flow Cytometry Core Facility, University of Helsinki. Untreated cells served as the negative control.

- 1. Lipid droplet assay

The cells were fixed with 4% formaldehyde in D-PBS with 0,9mM Ca^2+^ and 0,5mM Mg^2+^ at RT for 20 min. The unspecific binding sites were blocked with 5% normal goat serum in D-PBS at RT for 1 h. The iMGs were incubated with Iba1 (Supplementary materials, Table 2) primary antibody in 5% normal goat serum in D-PBS at 4°C overnight following secondary antibody (Supplementary materials, Table 2) incubation at RT for 1 h. Lipid droplets were visualized by LipidSpot^TM^ 488 Lipid Droplet Stain (#70065, Biotium, 1:1000) and nuclei were visualized by DAPI (Merck Millipore) staining in the dark at RT for 30min. The coverslips were washed once with D-PBS and leaved in D-PBS. Before imaging the coverslips were transferred to MatTek P35G-1.5-14-C 3,5 cm glass diameter dishes and D-PBS was added on top. The images were acquired with Andor Dragonfly 505 high speed spinning disc confocal microscope with 40x/APO LWD objective. The Lipid droplet quantification was conducted with Imaris (BITPLANE) using the Cells-function. Iba1 labeling was used to delineate the boundaries of the plasma membrane and to select for Lipid droplets inside the cells. The number of nuclei was measured separately using the Spots-function. The number of Lipid droplets was normalized to the number of nuclei. Per iPSC line per treatment 1-2 coverslips and 4 images per coverslip were quantified.

- 1. Cholesterol assay

The cells were grown and maturated on 6-well plates. On the day of the collection the medium was removed, 1ml of ice-cold PBS was added on top of the iMGs and the cells were scraped off on ice. The cells were centrifuged 5 min 1000G at 4°C, the supernatant was discarded, and the cells were transferred on dry ice and stored at -80°C until the day of the analysis. The cholesterol levels at basal conditions in iMGs were analyzed by using Amplex^TM^ Red Cholesterol Kit (#A12216, Invitrogen) according to the instructions provided by the manufacturer. The protein concentrations were measured using BCA kit (#10741395, Thermo Fisher Scientific). The cholesterol levels were normalized to 100μg/ml of protein.

- 1. Transwell migration assay

For transwell migration assay, the cells were grown in the iMG medium without maturation factors CD200 and CX3CL1. On the day of the assay, the transwell inserts (#83.3932.300, Sarstedt) with pore size 3 μm were placed inside 24-well plates containing basal medium (iMG medium without cytokines and DMEM/F12 without phenol red (#11039021, Gibco)) with or without chemoattractant ADP at a final concentration of 100 μM (#2574, Sigma) on the bottom of the wells. The iMGs were collected, washed, and plated in the basal medium at a density of 30,000 cells per transwell insert in a final volume of 100 μl. The cells on transwell inserts on top of 24-well plates were incubated for 4 h at 37°C following staining of the nuclei of the migrated iMGs on the bottom of the 24-well plates with Hoechst dye (#10150888, Fisher Scientific) at a final concentration of 1.25 μg/ml. After 30 min incubation at 37°C, the inserts were removed and images of the stained nuclei on the bottom of the wells were acquired with EVOS M5000 (Invitrogen by Thermo Fisher Scientific) at 4x magnification. The migrated nuclei were quantified with ImageJ Software.

**Table 3**. Primers used in RT-qPCR.

| **Primers** | **Producer** | **Catalog number** |
| --- | --- | --- |
| P2RY12 | Thermo Fisher Scientific | Hs00375457_m1 |
| TREM2 | Thermo Fisher Scientific | Hs00219132_m1 |
| APOE | Thermo Fisher Scientific | Hs00171168_m1 |
| MSR1 | Thermo Fisher Scientific | Hs00234007_m1 |
| TLR2 | Thermo Fisher Scientific | Hs00152932_m1 |
| TLR4 | Thermo Fisher Scientific | Hs00152939_m1 |
| CLEC7A | Thermo Fisher Scientific | Hs00224028_m1 |
| MARCO | Thermo Fisher Scientific | Hs00198937_m1 |
| CTSD | Thermo Fisher Scientific | Hs00157205_m1 |
| CTSB | Thermo Fisher Scientific | Hs00947439_m1 |
| MAP1LC3B | Thermo Fisher Scientific | Hs00917683_m1 |
| FABP3 | Thermo Fisher Scientific | Hs07287863_m1 |
| LPL | Thermo Fisher Scientific | Hs00173425_m1 |
| AGPAT4 | Thermo Fisher Scientific | Hs0108853_m1 |
| FAR1 | Thermo Fisher Scientific | Hs00386153_m1 |
| PDK4 | Thermo Fisher Scientific | Hs01037712_m1 |
| GCLC | Thermo Fisher Scientific | Hs00155249_m1 |
| IL1B | Thermo Fisher Scientific | Hs01555410_m1 |
| SLC1A5 | Thermo Fisher Scientific | Hs01056542_m1 |
| SLC7A5 | Thermo Fisher Scientific | Hs01001189_m1 |
| GAPDH | Thermo Fisher Scientific | Hs99999905_m1 |
| ACTB | Applied Biosystems | 4326315E |


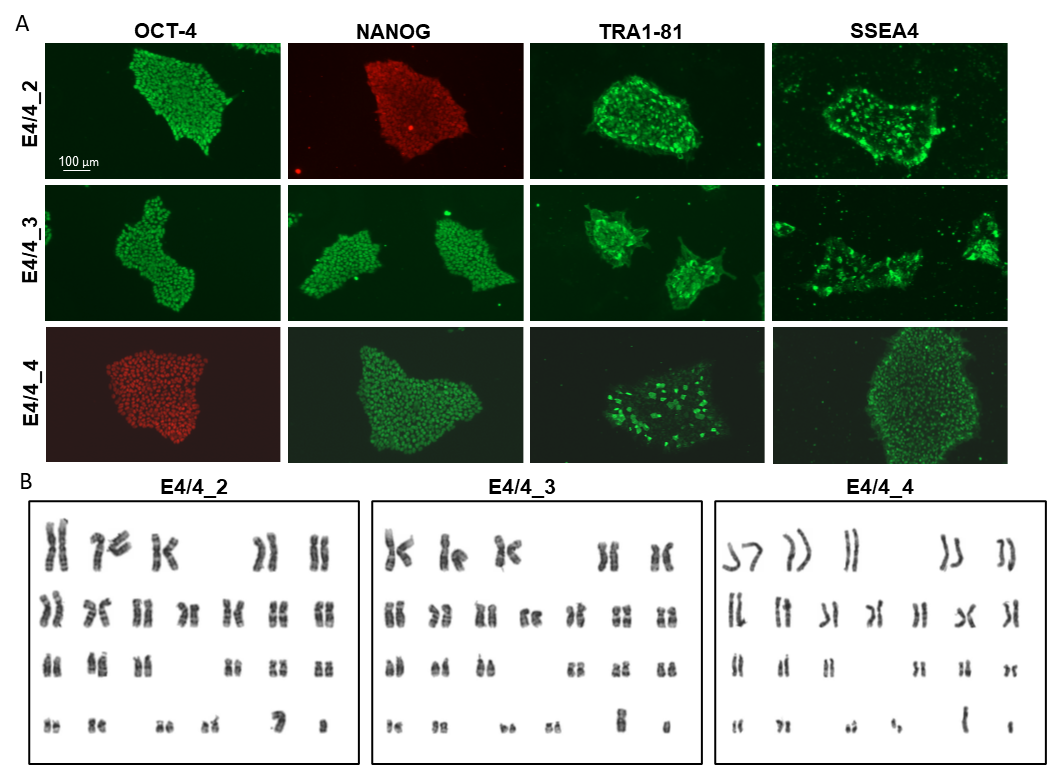


**Supplementary Figure 1**. **Characterization of previously unpublished iPSC lines.** Immunocytochemistry images of the expression of pluripotency markers octamer-binding transcription factor 4 (OCT-4), NANOG, TRA1-81 and stage-specific embryonic antigen 4 (SSEA-4) in E4/4_2, E4/4_3 and E4/4_4 iPSC colonies. Scale bar 100 μm. B, karyograms of E4/4_2, E4/4_3, E4/4_4, E3/3_2_TdTomato and E4/4_4_TdTomato iPSC lines showing normal 46 XY karyotypes. The cells were karyotyped by Ambar Lab, Barcelona, Spain.


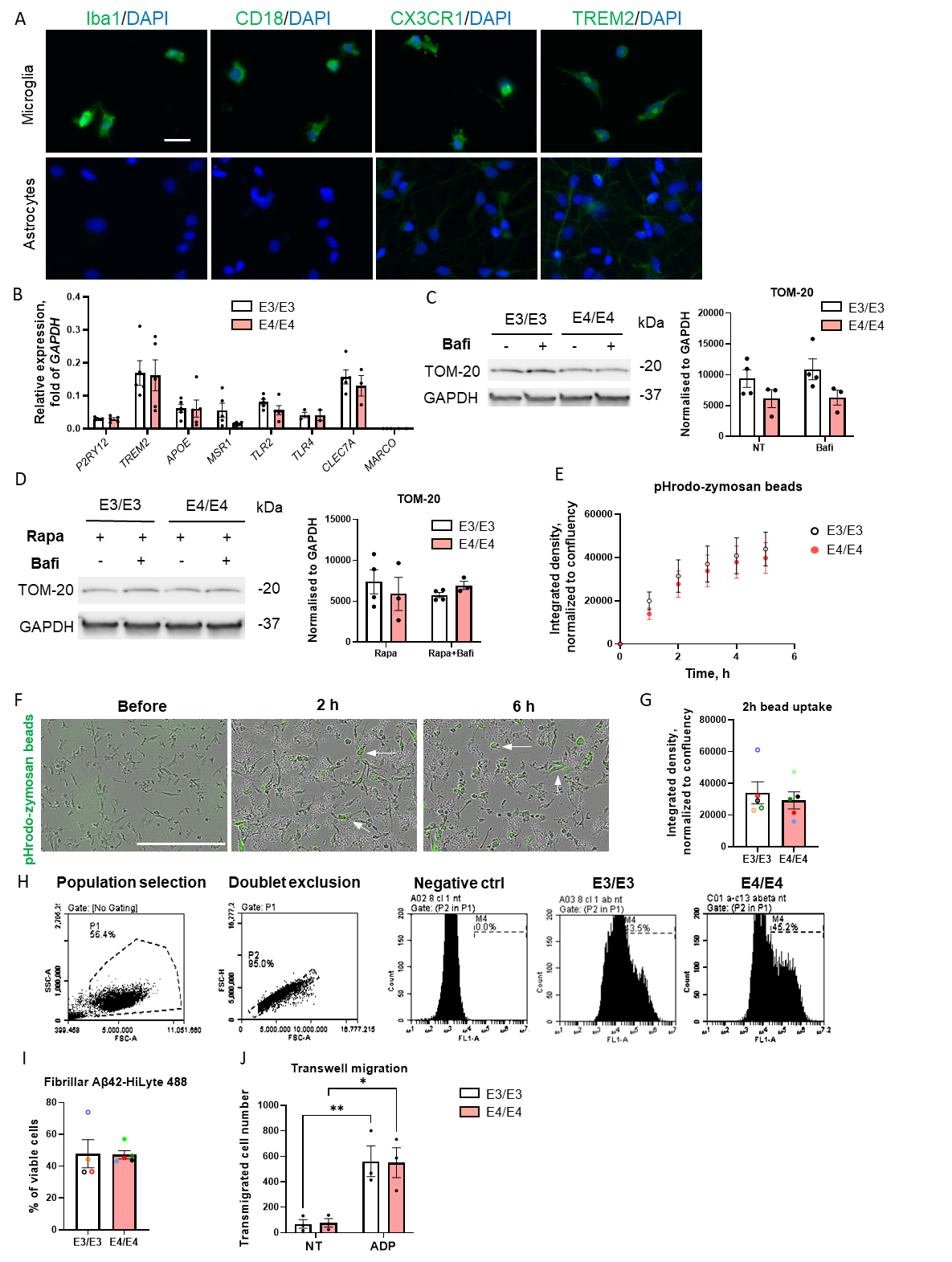


**Supplementary Figure 2. Characterization of E3/E3 and E4/E4 iMGs.** A, iMGs are immunoreactive for Iba1, CD18, CX3CR1 and TREM2. iPSC-derived astrocytes are used as a negative control. Scale bar 50 μm. B, both E3/3 and E4/4 iMGs express microglia-associated genes and there are no significant differences between the genotypes. C, D, representative Western Blot images and the quantification analyses of TOM-20 in iMGs with (+) or without (-) rapamycin (rapa) and bafilomycin A (bafi) treatment. E, the uptake of pHrodo-conjugated zymosan-coated beads assessed using Incucyte S3. N = 5 iPSC lines per genotype. F, representative images of iMGs phagocytosing pHrodo-conjugated beads. The cells that have internalized beads look green (arrows). Scale bar 300 μm. G, I, the quantification of pHrodo-conjugated bead (G) and fibrillar Aβ42 (I) uptake at 2h. H, the flow cytometry gating strategy for the quantification of fibrillar Aβ42 uptake, HiLyte^TM^ Fluor 488-labeled Aβ42 fibrils taken up by iMGs emitted fluorescence in FL1-A channel. FSC-A, forward scatter area; SSC-A, side scatter area; FSC-H, forward scatter height. J, the quantification of migrated iMGs with or without chemoattractant ADP at 4h. P value is derived from two-way repeated measures ANOVA. *, p < 0.05. **, p < 0.01. Individual dots in the bar plots represent the mean values for individual iPSC lines. The color coding of iPSC lines is shown in Supplementary table 1. Data in the graphs are shown as mean ± SEM.

**
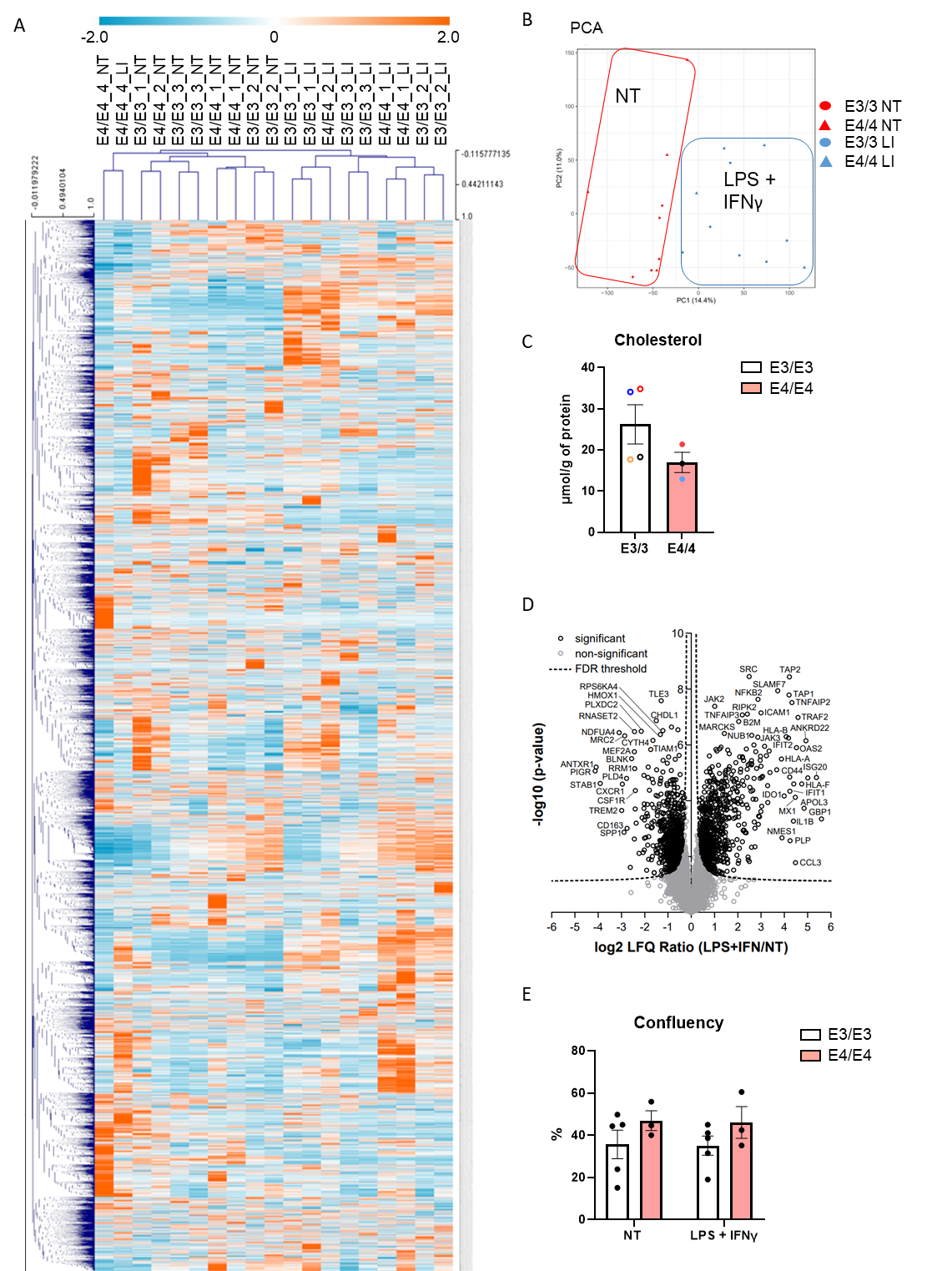
**

**Supplementary Figure 3.**  **Metabolism, cholesterol levels and confluency in iMGs.** A, Heat map of the normalized signal abundances of the detected molecular features with signal-to-noise ratio > 5 (n = 16 318) in each studied sample. A hierarchical clustering was applied to arrange the metabolites, and the samples based on their similarity of the abundance between the samples. The color represents the z-score of the abundance, i.e. the number of standard deviations above (positive value, red color) or below (negative value, blue color) the mean abundance of that molecular feature in all samples. NT, no treatment (basal conditions); LI, LPS + IFNγ treatment. B, Principal component analysis (PCA) of the metabolite profiles of each sample based on all detected molecular features (n = 26 881). Component 1 (x axis) explains 14.4% of the variation between the samples and component 2 (y axis) explains 11.0% of the between-samples variation. C, the quantification of cholesterol levels in iMGs at basal conditions. D, Volcano plot of comparing LPS and IFN treated cells with non-treated cells (n=5). The minus log10 transformed p-values are plotted against the log2 transformed fold changes for each protein. The threshold of a permutation-based FDR correction for multiple hypotheses is indicated as dashed lines. Proteins with a significant change are indicated as black circles whereas non-significantly altered proteins are indicated as gray circles. Selected proteins are labeled with their UniProt gene names. E, the quantification of confluency of iMGs treated for 24h with LPS+IFNγ or left untreated (NT). The confluency was assessed using Incucyte S3. Dots represent individual iPSC lines. The color coding of iPSC lines is shown in Supplementary table 1. Data in the graphs are shown as mean ± SEM.

**
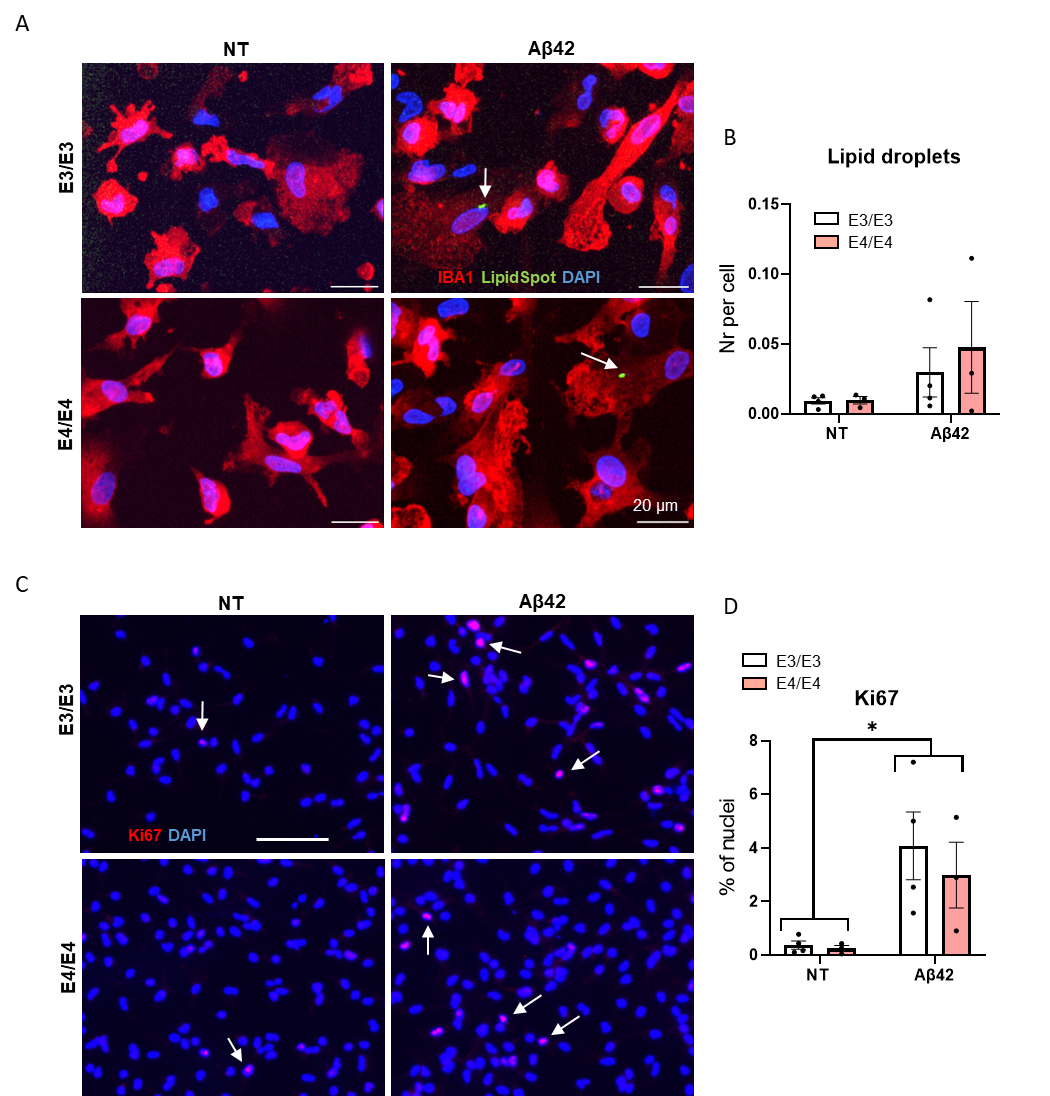
**

**Supplementary Figure 4. Lipid droplet levels and proliferation rate in iMGs.** A-B, representative images of LipidSpot+ lipid droplets in the cytoplasm of Iba1+ iMGs (A) and the quantification data of lipid droplets (B) in iMGs treated with Aβ42 or left untreated (NT). Scale bar 20μm. Arrows, iMGs with lipid droplets. C, representative images of Ki67+ nuclei (arrows). Scale bar 100 μm. D, percentages of Ki67+ nuclei in iMGs treated for 48h with Aβ42 or left untreated (NT). P value is derived from two-way repeated measures ANOVA. *, p < 0.05. Dots represent individual iPSC lines. Data in the graphs are shown as mean ± SEM.

**References**

1. Kettunen, P., et al., *SARS-CoV-2 Infection of Human Neurons Is TMPRSS2 Independent, Requires Endosomal Cell Entry, and Can Be Blocked by Inhibitors of Host Phosphoinositol-5 Kinase.* J Virol, 2023. **97**(4): p. e0014423.

2. Jantti, H., et al., *Microglial amyloid beta clearance is driven by PIEZO1 channels.* J Neuroinflammation, 2022. **19**(1): p. 147.

3. Schmid, B., et al., *Generation of a set of isogenic iPSC lines carrying all APOE genetic variants (E2/E3/E4) and knock-out for the study of APOE biology in health and disease.* Stem Cell Res, 2021. **52**: p. 102180.

4. Takalo, M., et al., *The protective PLCgamma2-P522R variant mitigates Alzheimer's disease-associated pathologies by enhancing beneficial microglial functions.* J Neuroinflammation, 2025. **22**(1): p. 64.

5. Wu, Y.C., et al., *Human iPSC-derived pericyte-like cells carrying APP Swedish mutation overproduce beta-amyloid and induce cerebral amyloid angiopathy-like changes.* Fluids Barriers CNS, 2024. **21**(1): p. 78.
