# Supplementary material for: Inflammation-induced lysosomal dysfunction in human iPSC-derived microglia is exacerbated by APOE 4/4 genotype
Source: J Neuroinflammation. 2025 Jun 2;22:147. doi: 10.1186/s12974-025-03470-y (PMC12131611; doi:10.1186/s12974-025-03470-y)
Supplement: Supplementary file 3 — Supplementary Material 3 [file 12974_2025_3470_MOESM3_ESM.pdf]

Blots for LC3A/B I/II and GAPDH expression quantification (Figure 1 B) Batch 2021

Merge with ladder images

Images for quantification

LC3A/B I/II Batch 2021

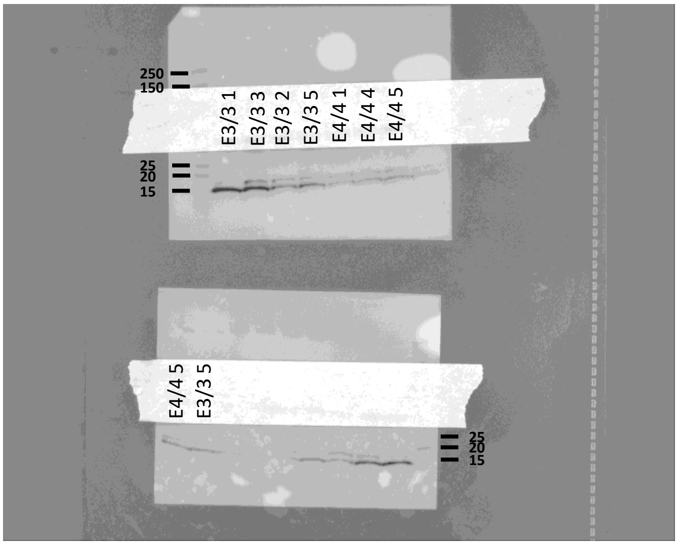

LC3A/B I/II 14,16 kDa

LC3A/B I/II Batch 2021

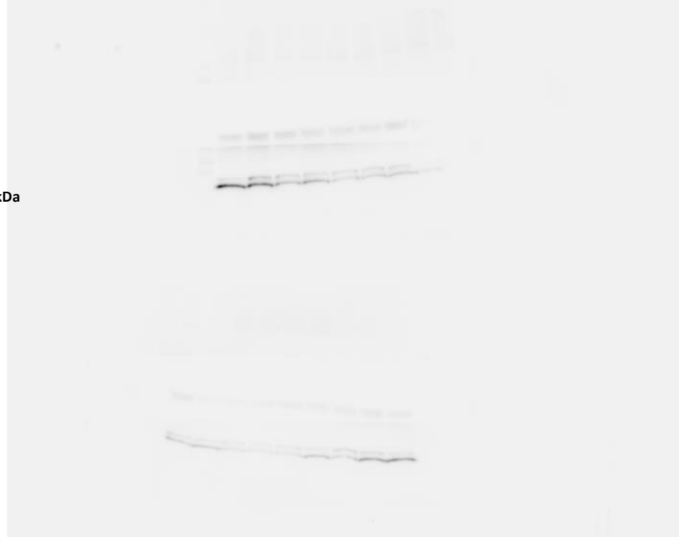

GAPDH Batch 2021

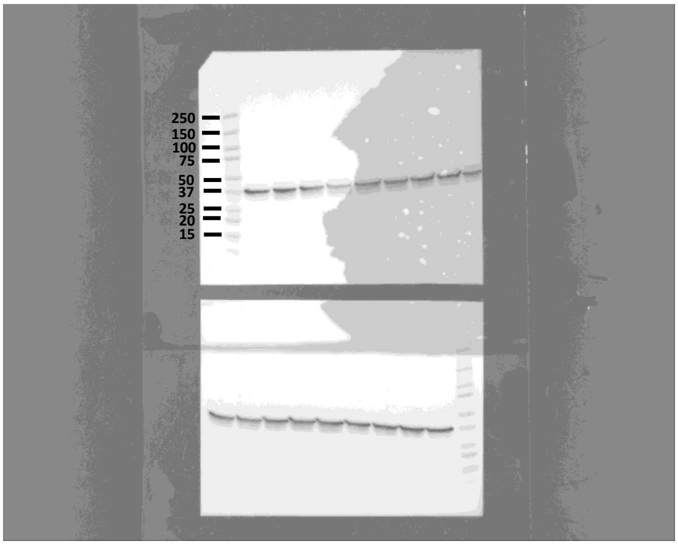

GAPDH 37 kDa

GAPDH Batch 2021

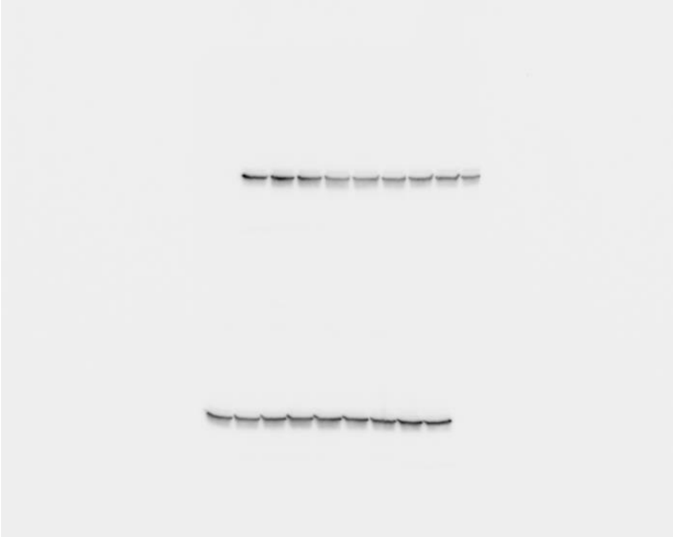

Blots for LC3A/B I/II , TOM-20 and GAPDH expression quantification (Figure 1 B, D; Supplementary Fig. 2C)

Batch 2023

Merge with ladder images

Images for quantification

LC3A/B I/II Batch 2023, set 2

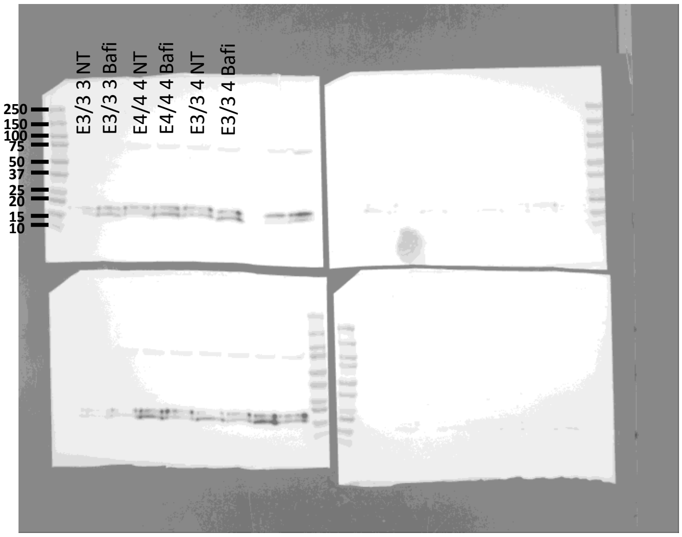

LC3A/B I/II Batch 2023, set 2

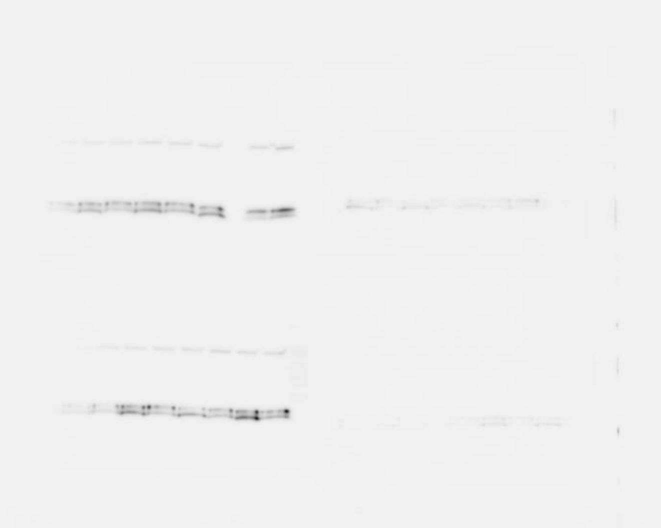

LC3A/B I/II 14,16 kDa

TOM-20 Batch 2023, set 2

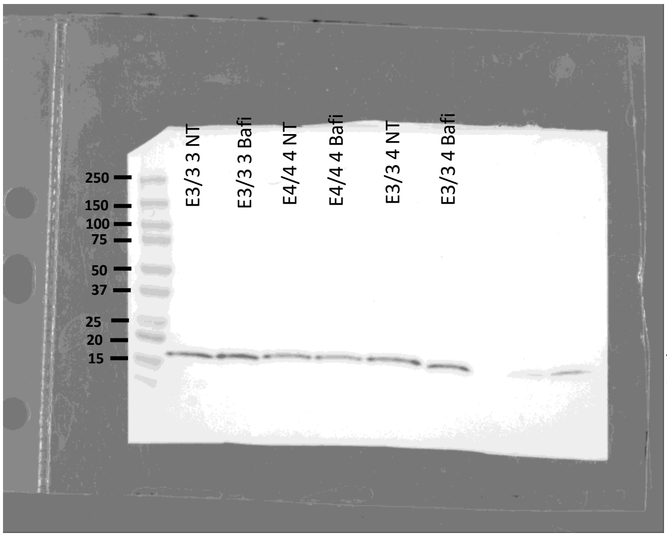

TOM-20 Batch 2023, set 2

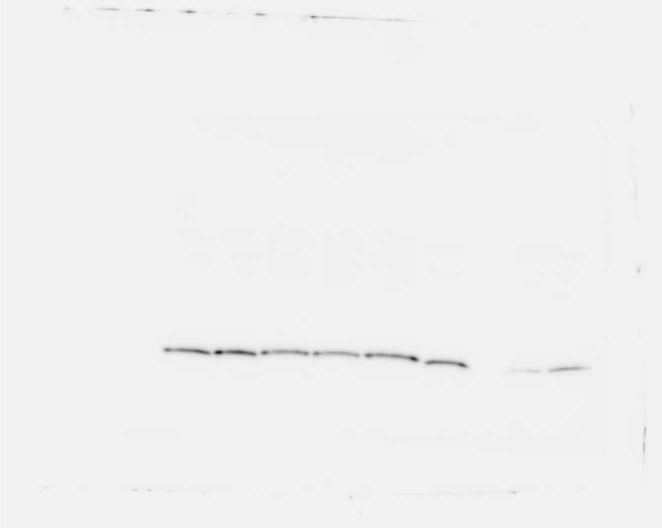

TOM-20 20 kDa

GAPDH Batch 2023, set 2

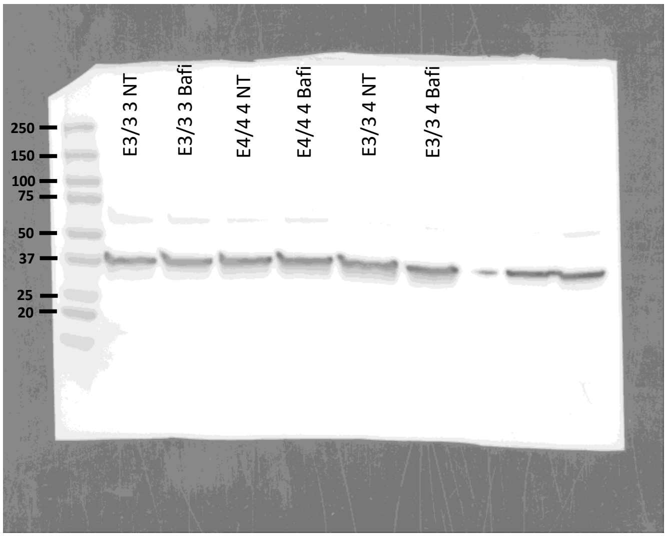

GAPDH Batch 2023, set 2

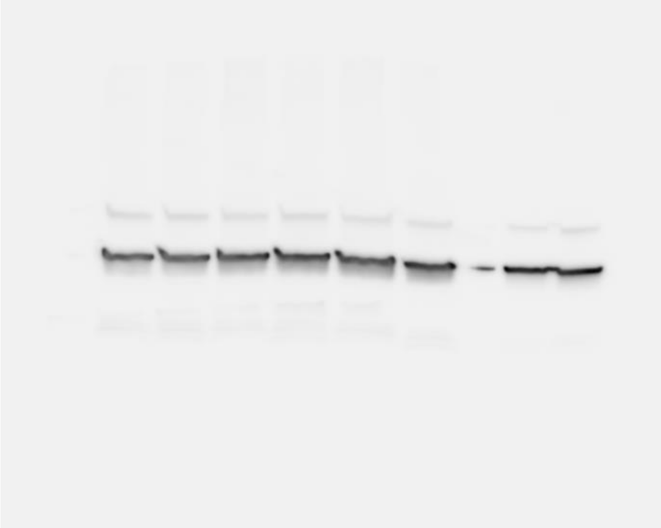

GAPDH 37 kDa

Blots for LC3A/B I/II, TOM-20 and GAPDH expression quantification (Figure 1 B, D; Supplementary Fig. 2C)
Batch 2023

Merge with ladder images

Images for quantification

LC3A/B I/II Batch 2023, set 2

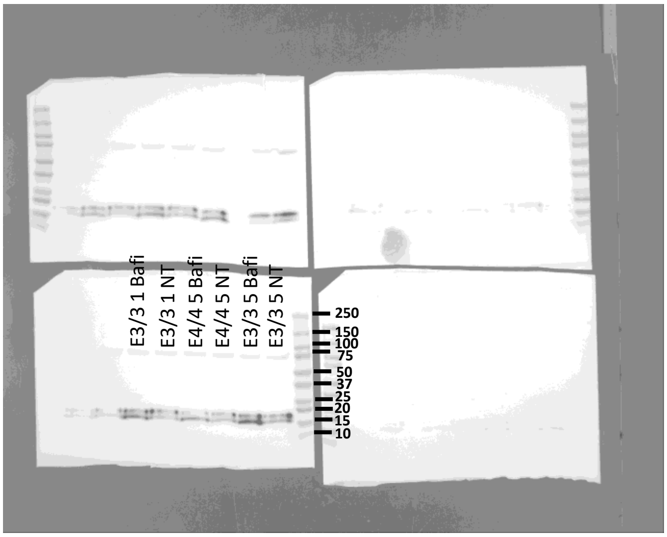

LC3A/B I/II Batch 2023, set 2

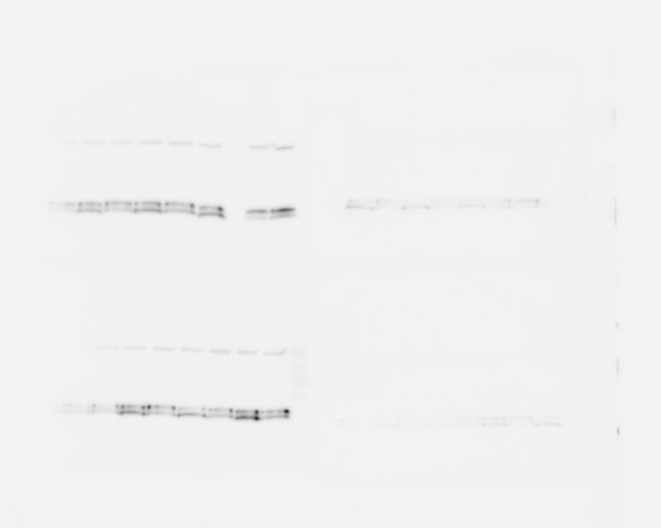

LC3A/B I/II 14,16 kDa

TOM-20 Batch 2023, set 2

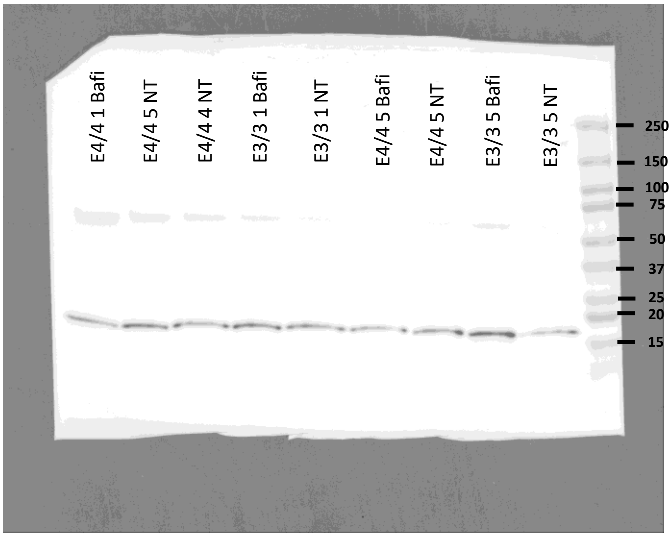

TOM-20 Batch 2023, set 2

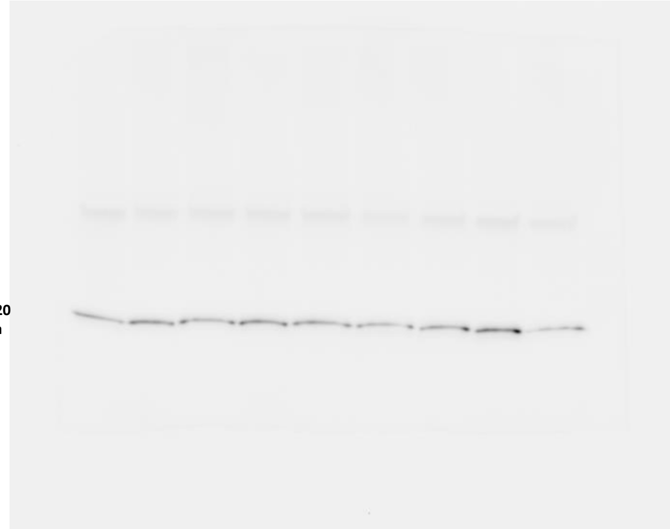

TOM-20 20 kDa

GAPDH Batch 2023, set 2

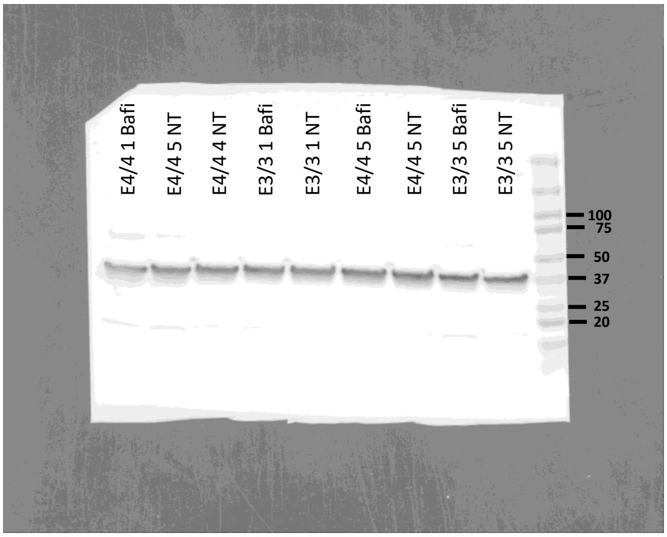

GAPDH Batch 2023, set 2

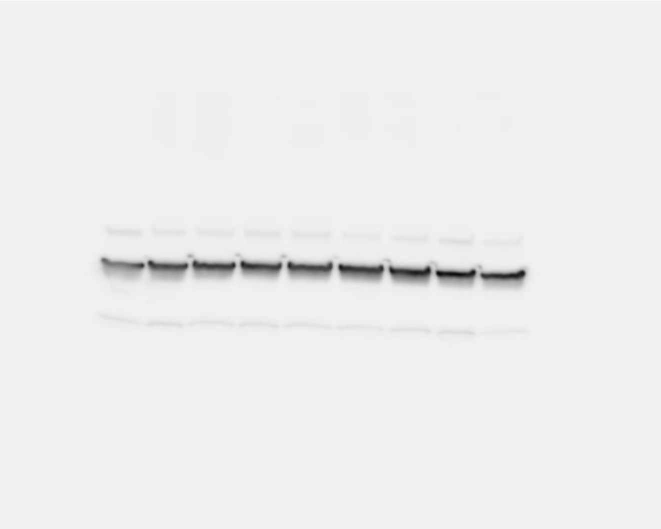

GAPDH 37 kDa

Blots for LC3A/B I/II, TOM-20 and GAPDH expression quantification (Figure 1 B, D, E; Supplementary Fig. 2C,D)
Batch 2023

Merge with ladder images

LC3A/B I/II Batch 2023, set 2

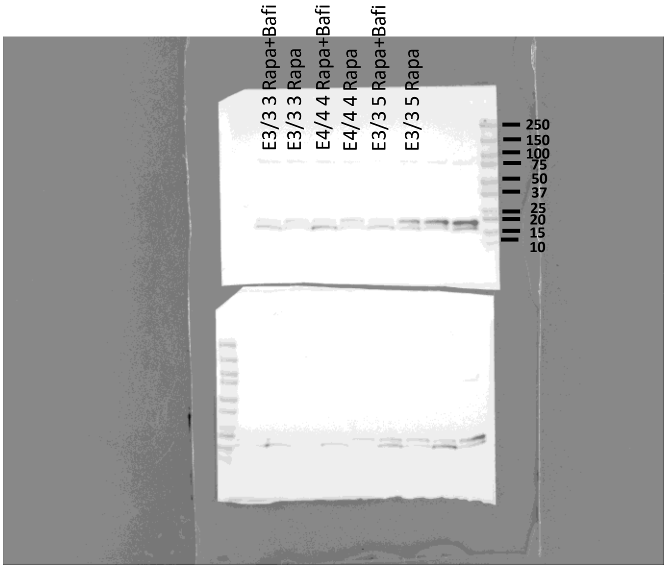

LC3A/B I/II 14,16 kDa

Images for quantification

LC3A/B I/II Batch 2023, set 2

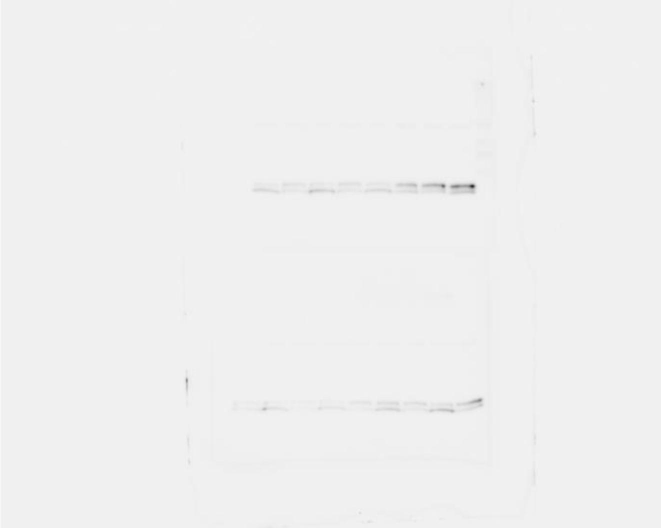

TOM-20 Batch 2023, set 2

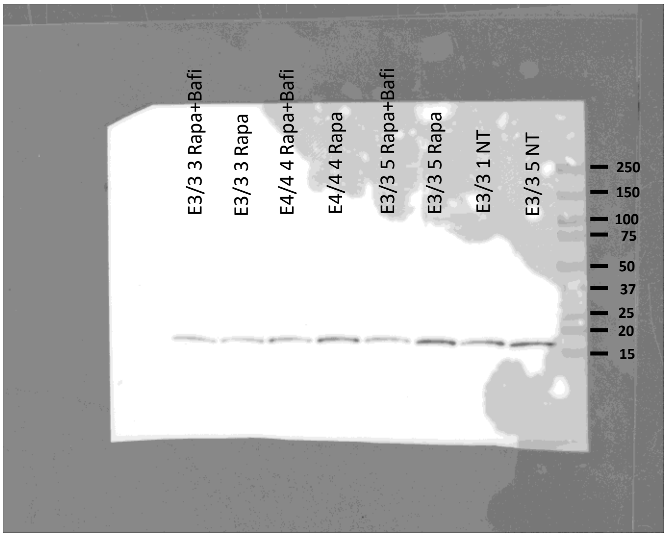

TOM-20 20 kDa

TOM-20 Batch 2023, set 2

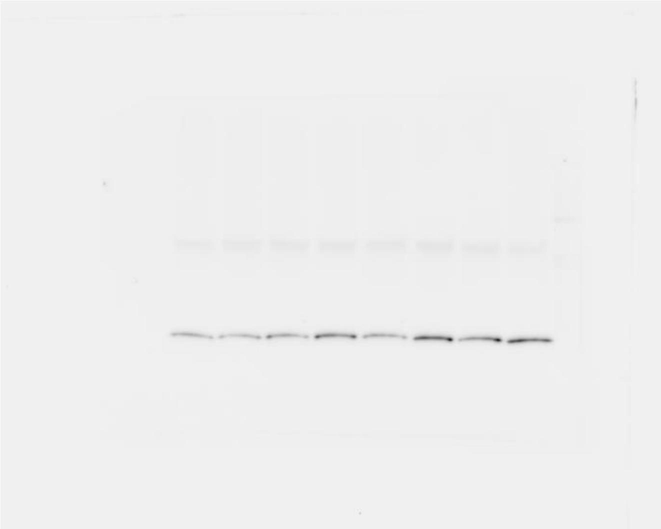

GAPDH Batch 2023, set 2

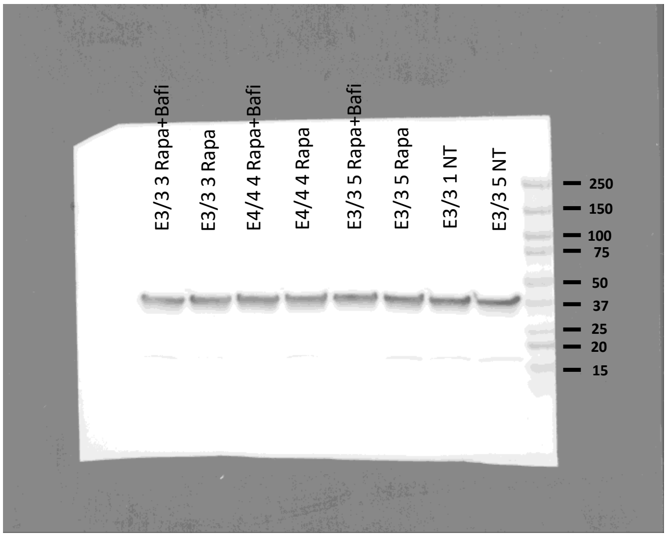

GAPDH 37 kDa

GAPDH Batch 2023, set 2

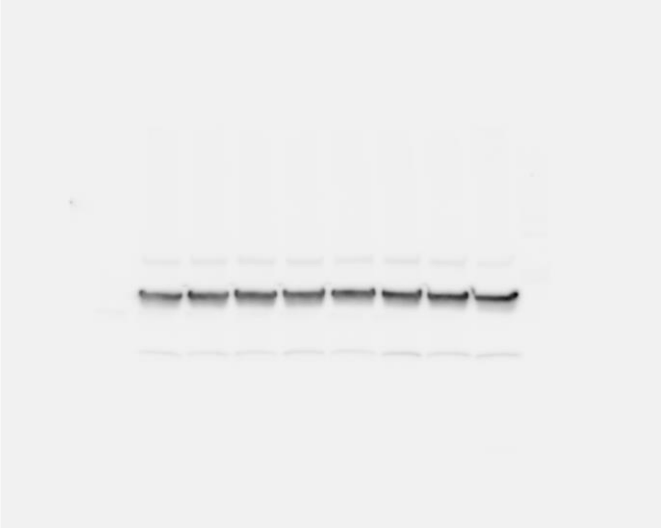

Blots for LC3A/B I/II, TOM-20 and GAPDH expression quantification (Figure 1 B, D, E; Supplementary Fig. 2C, D)
Batch 2023

Merge with ladder images

LC3A/B I/II Batch 2023, set 2

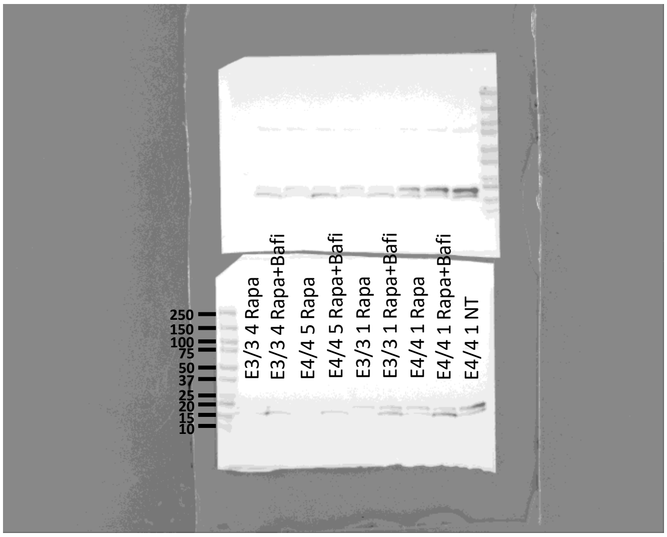

LC3A/B I/II 14,16 kDa

Images for quantification

LC3A/B I/II Batch 2023, set 2

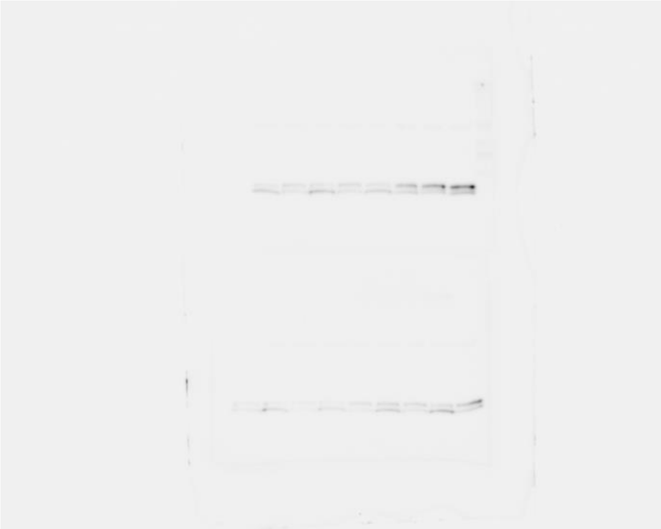

TOM-20 Batch 2023, set 2

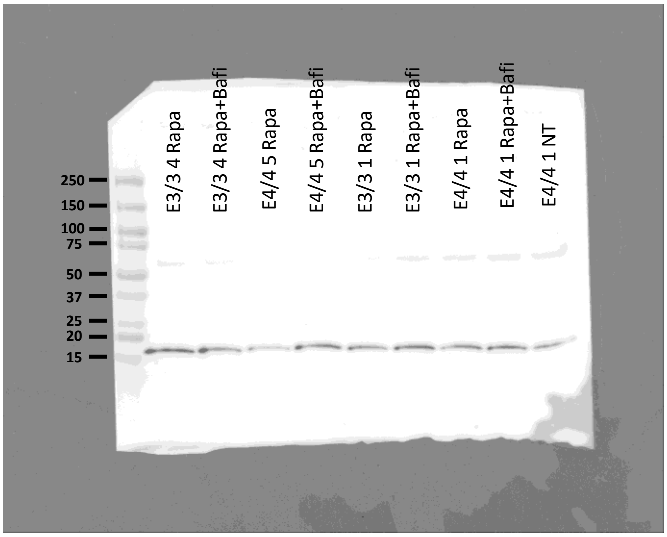

TOM-20 20 kDa

TOM-20 Batch 2023, set 2

GAPDH Batch 2023, set 2

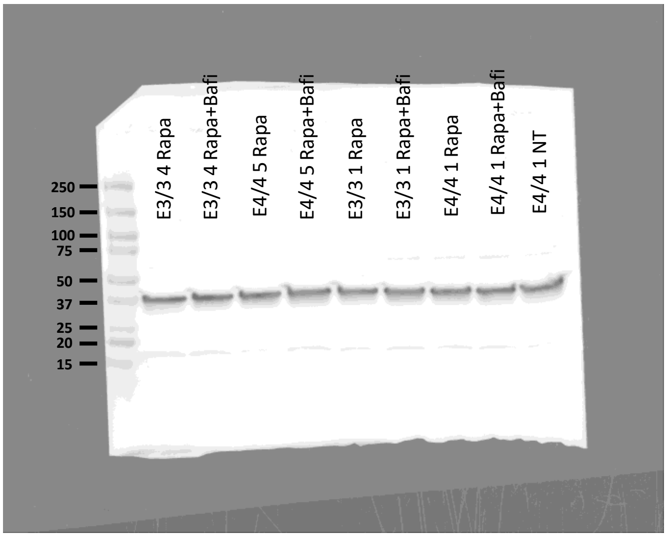

GAPDH 37 kDa

GAPDH Batch 2023, set 2

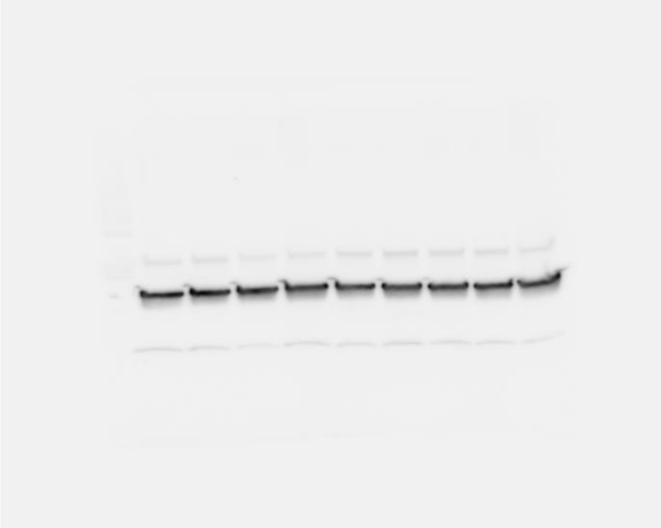

Blots for LC3A/B I/II and GAPDH expression quantification (Figure 1 B, D, E) Batch 2023

Merge with ladder images

Images for quantification

LC3A/B I/II Batch 2023, set 1

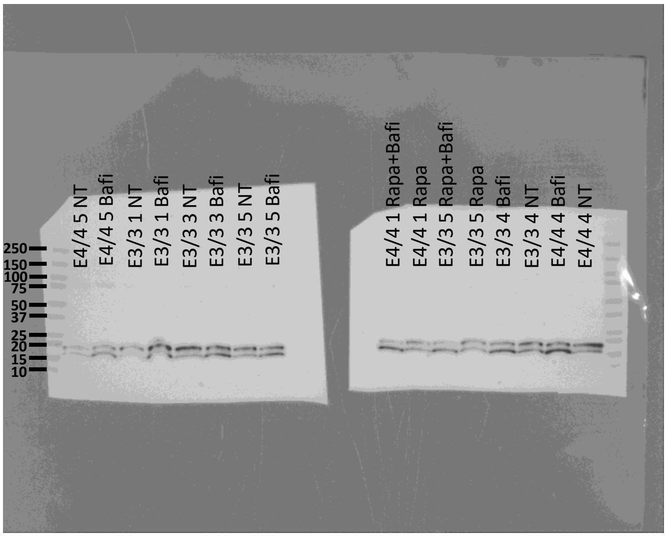

LC3A/B I/II Batch 2023, set 1

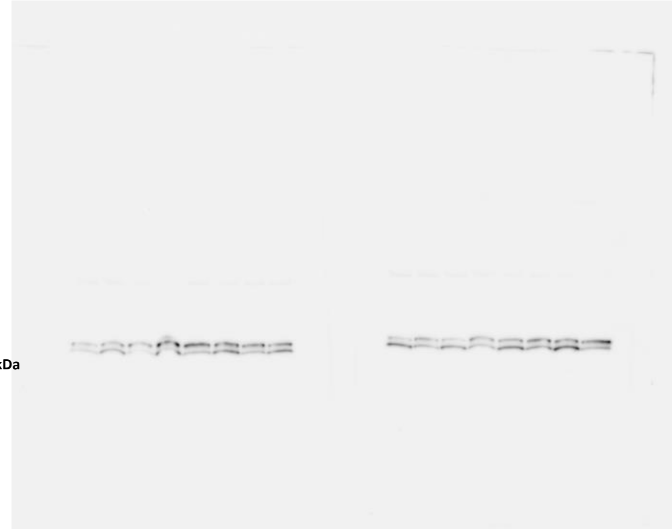

LC3A/B I/II 14,16 kDa

GAPDH Batch 2023, set 1

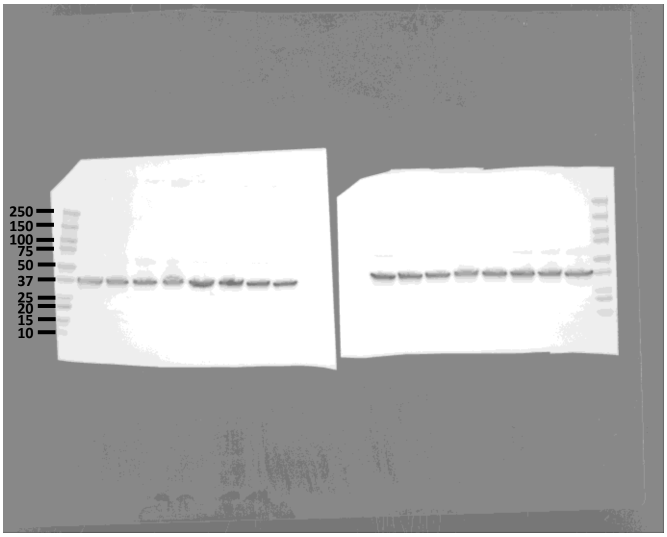

GAPDH Batch 2023, set 1

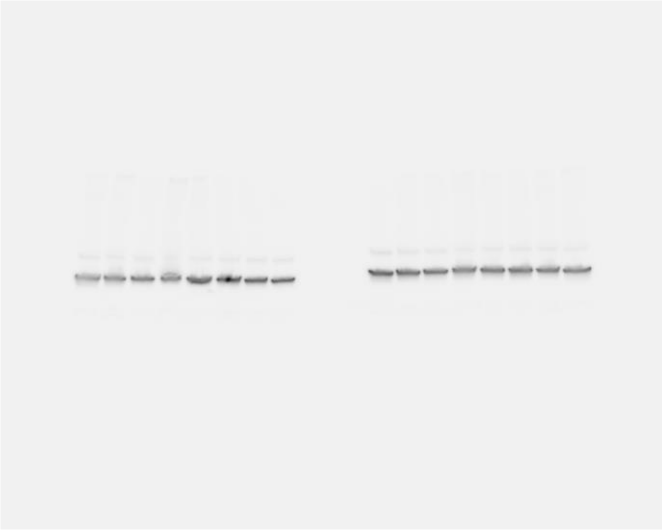

GAPDH 37 kDa

Blots for LC3A/B I/II and GAPDH expression quantification (Figure 1 B, D, E) Batch 2023

Merge with ladder images

LC3A/B I/II Batch 2023, set 1

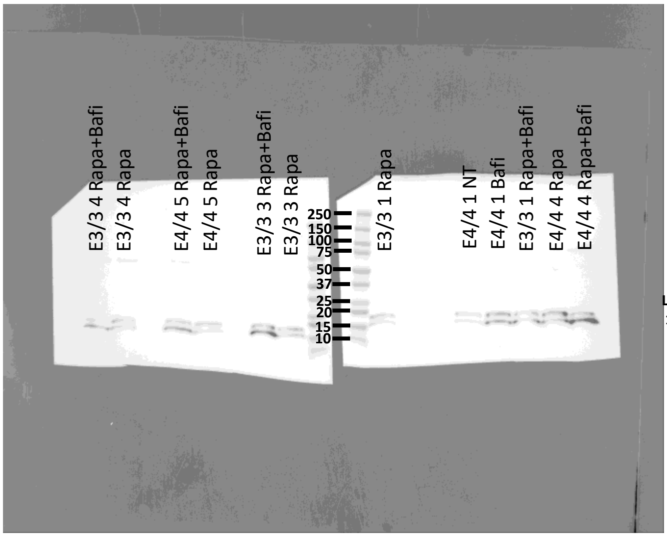

LC3A/B I/II
14, 16 kDa

TOM-20 Batch 2023, set 1

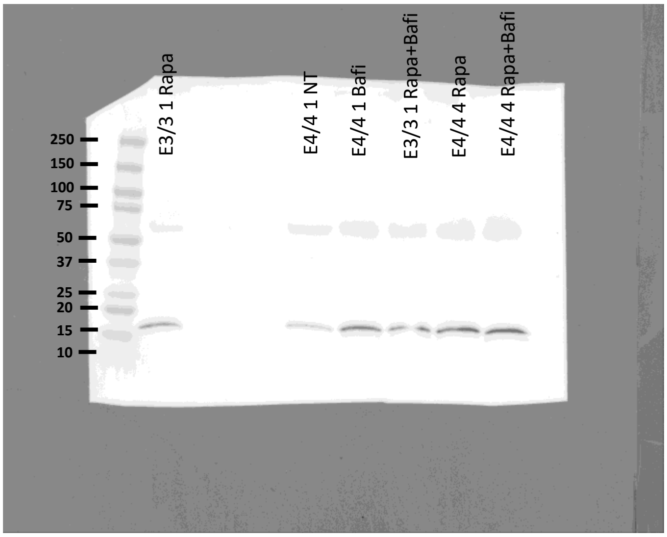

TOM-20
20 kDa

GAPDH Batch 2023, set 1

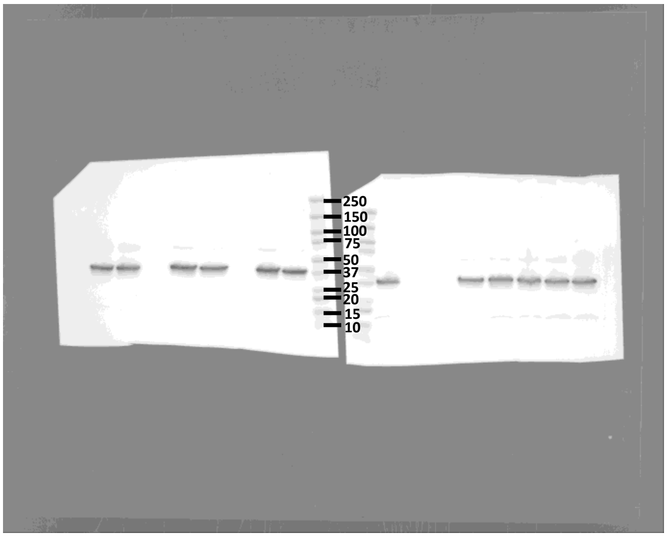

GAPDH
37 kDa

Images for quantification

LC3A/B I/II Batch 2023, set 1

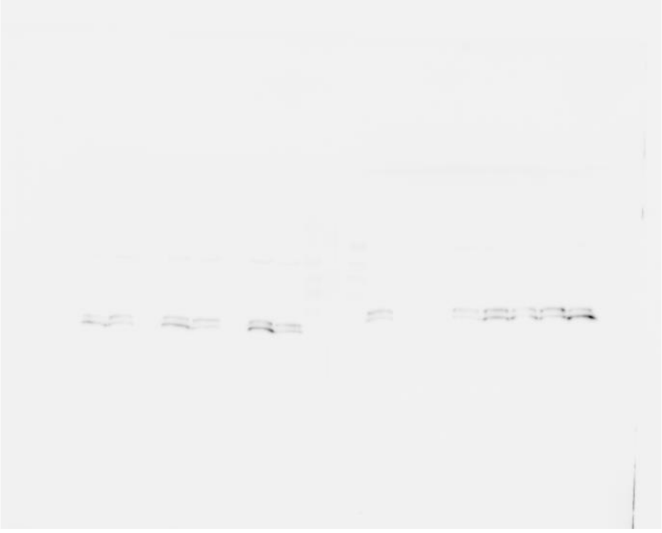

TOM-20 Batch 2023, set 1

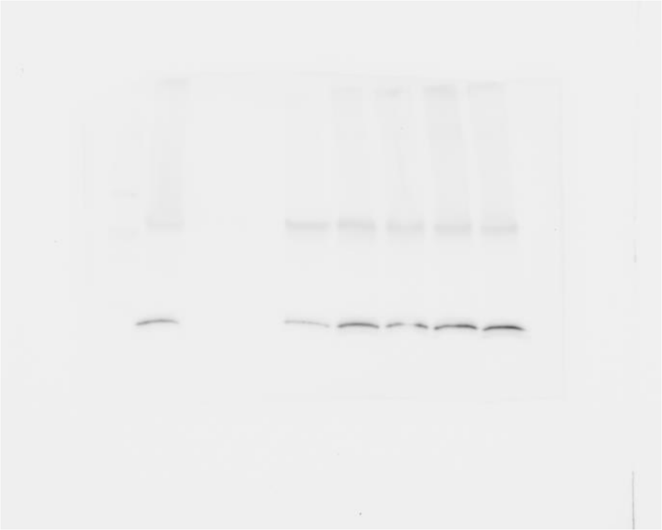

GAPDH Batch 2023, set 1

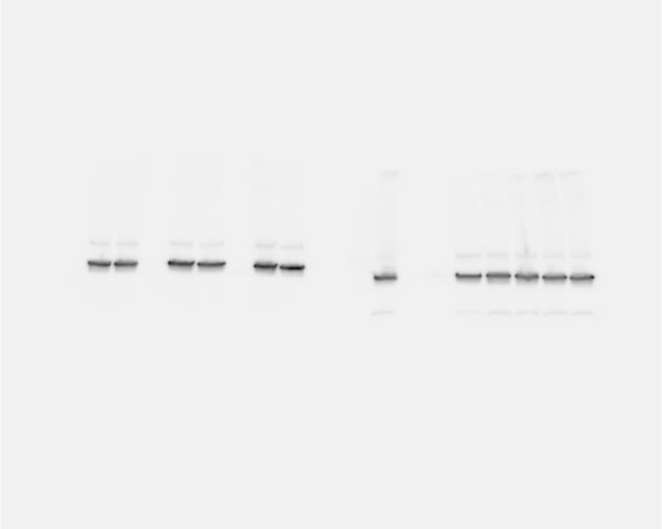

Blots for LC3A/B I/II and GAPDH expression quantification (Figure 1 B) Batch 2023

Merge with ladder images

Images for quantification

LC3A/B I/II Batch 2023, (19.2.2024)

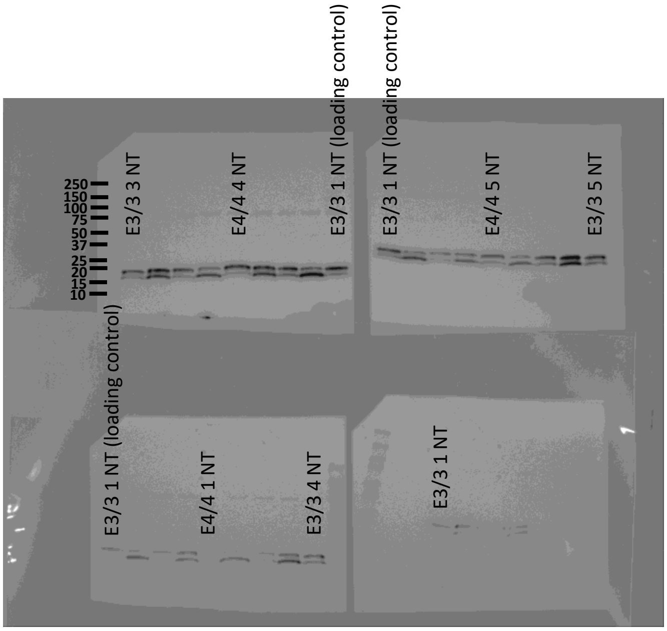

LC3A/B I/II Batch 2023

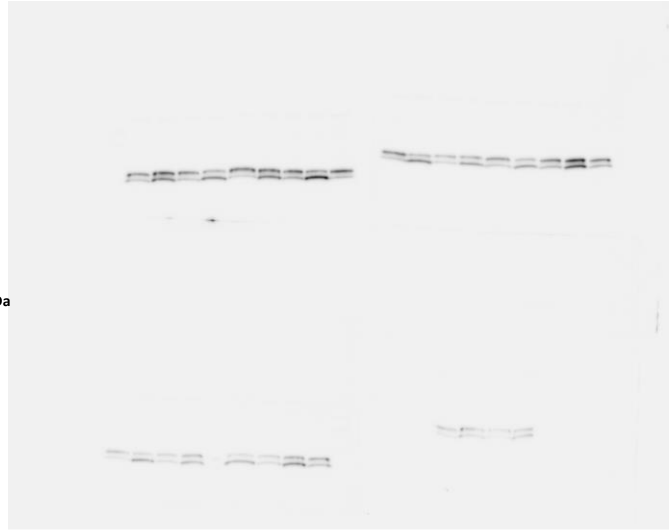

LC3A/B I/II 14,16 kDa

GAPDH Batch 2023

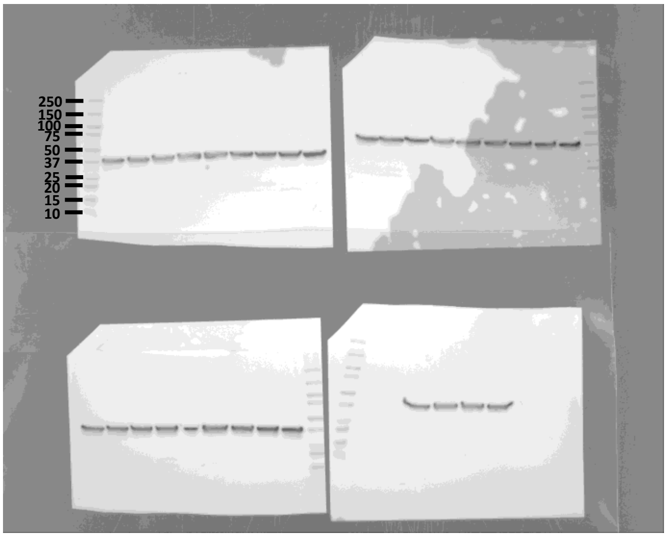

GAPDH Batch 2023

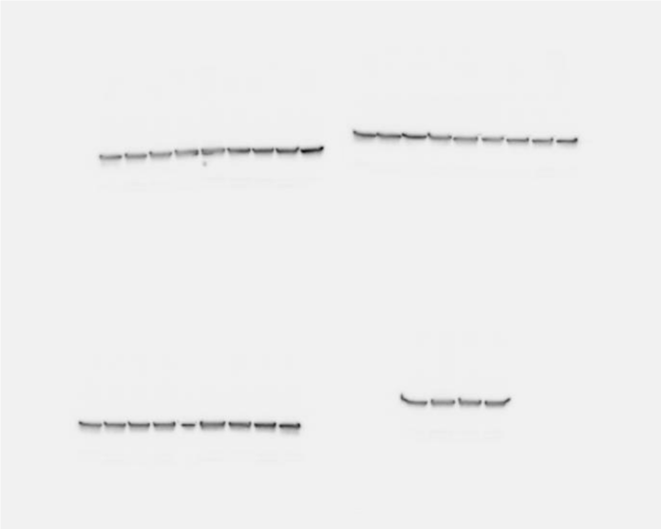

GAPDH 37 kDa

Blots for LC3A/B I/II and GAPDH expression quantification (Figure 1 B) Batch 2023

Merge with ladder images

Images for quantification

LC3A/B I/II Batch 2023 (25.10.23)

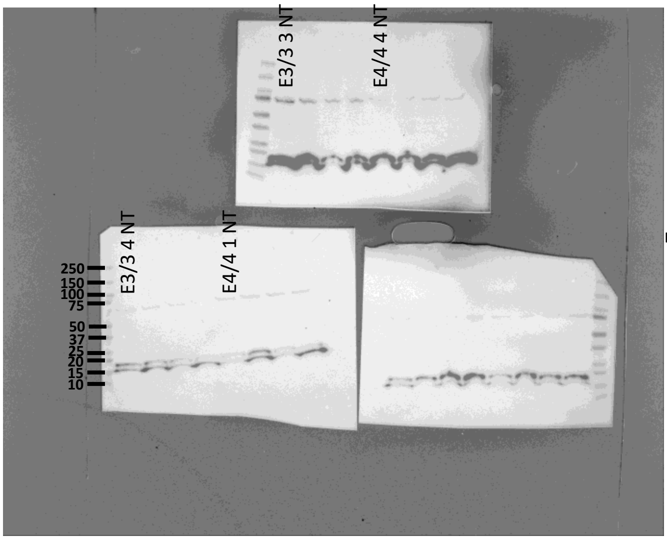

LC3A/B I/II Batch 2023

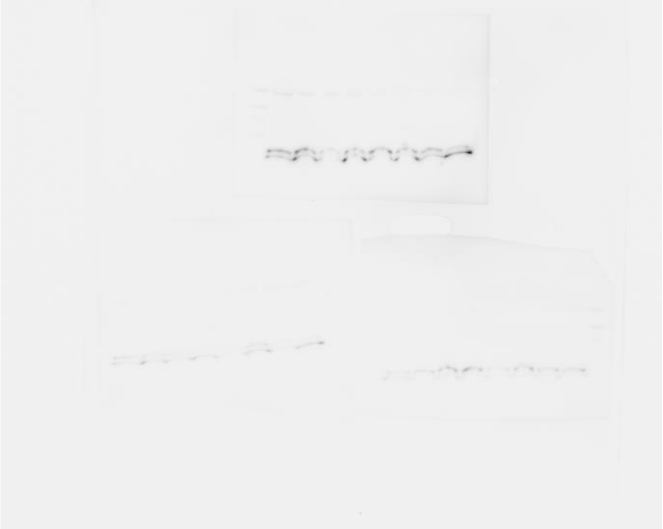

LC3A/B I/II
14, 16 kDa

GAPDH Batch 2023

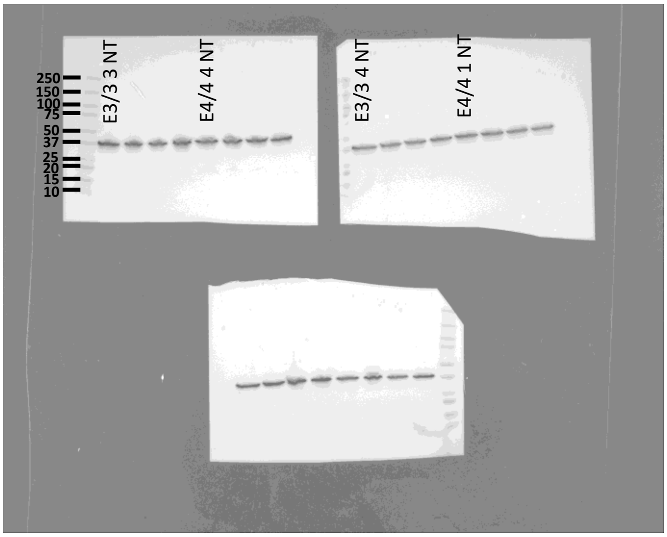

GAPDH Batch 2023

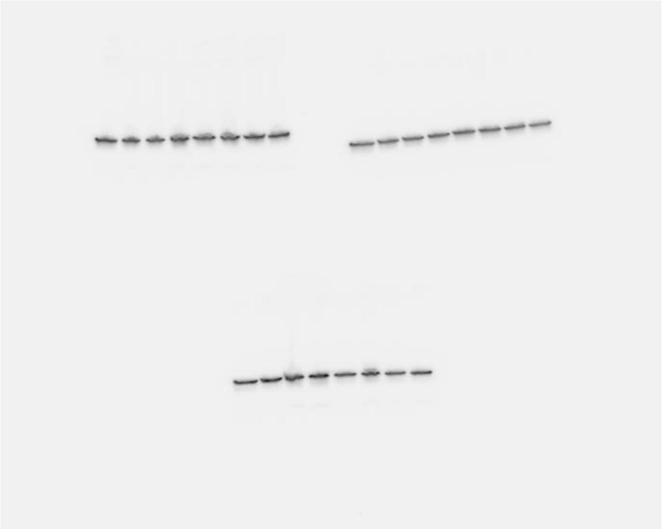

GAPDH
37 kDa

Blots for GLUL and LC-3 expression quantification after LPS+IFNγ treatment (Figure 3 C, D) Batch 2021

Merge with ladder images

LC3A/B Batch 2021

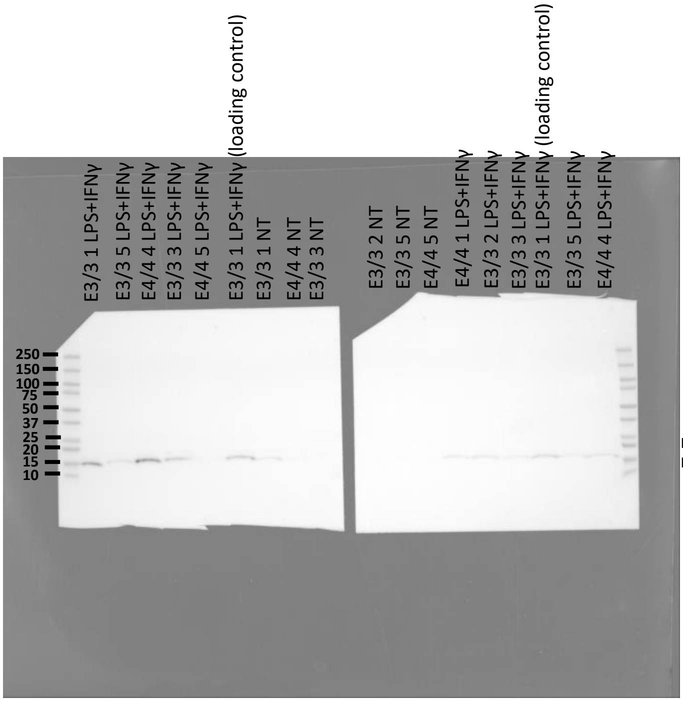

LC3A/B I/II 14,16 kDa

GLUL Batch 2021

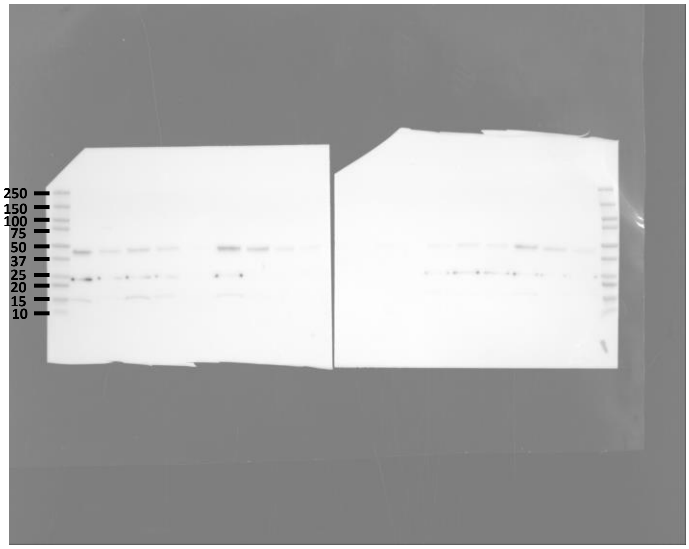

GLUL 42 kDa

β-actin Batch 2021

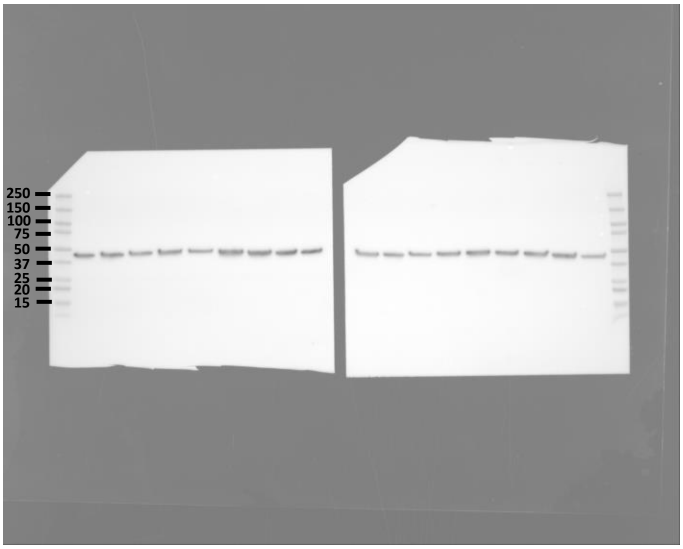

β-actin 45 kDa

Images for quantification

LC3A/B Batch 2021

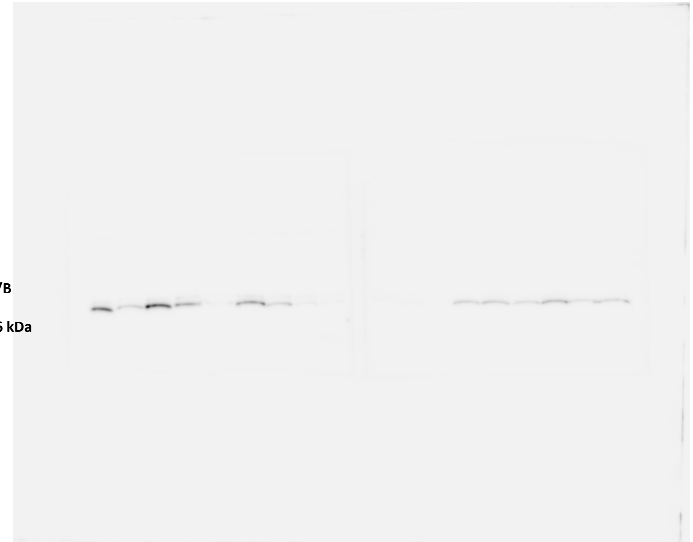

GLUL Batch 2021

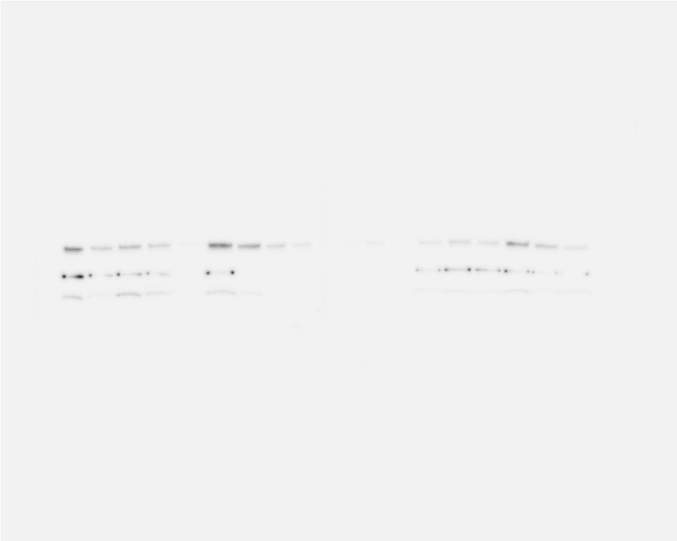

β-actin Batch 2021

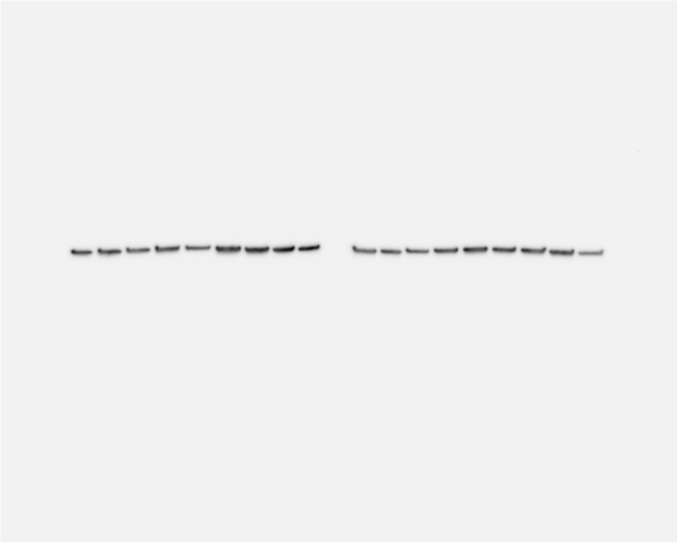

Blots for SQSTM1/p62 and LAMP2 expression quantification after Aβ42 treatment (Figure 4 C) Batch 2023

Merge with ladder images

SQSTM1/p62 Batch 2023, APOE3

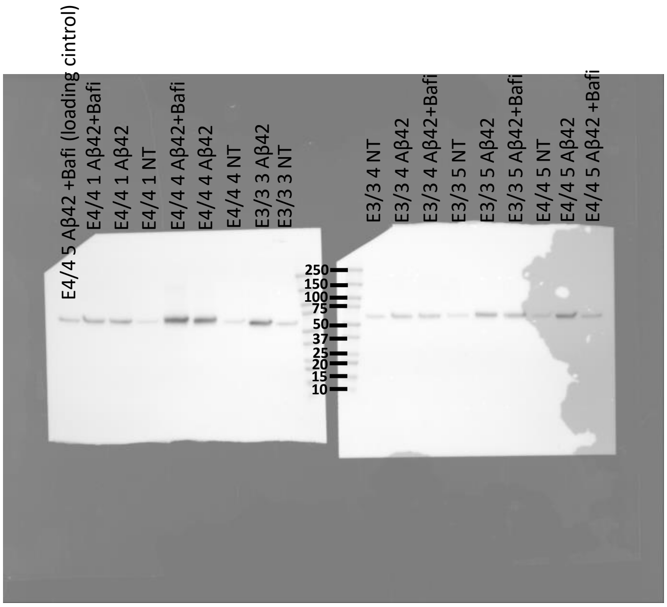

Images for quantification

SQSTM1/p62 Batch 2023

SQSTM1/
p62
62kDa

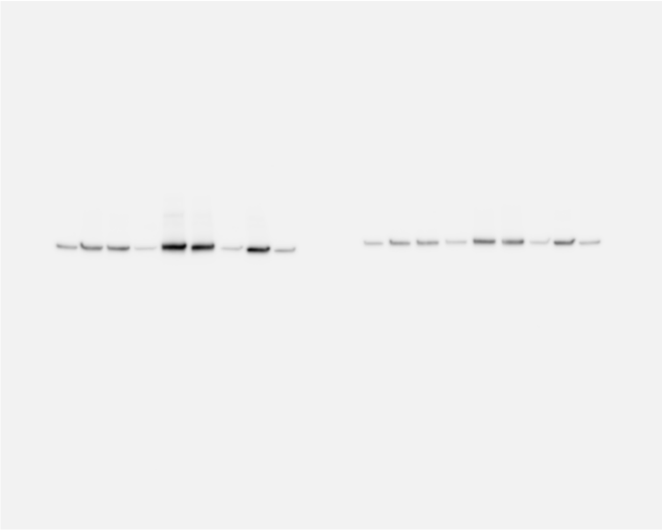

LAMP2 Batch 2023

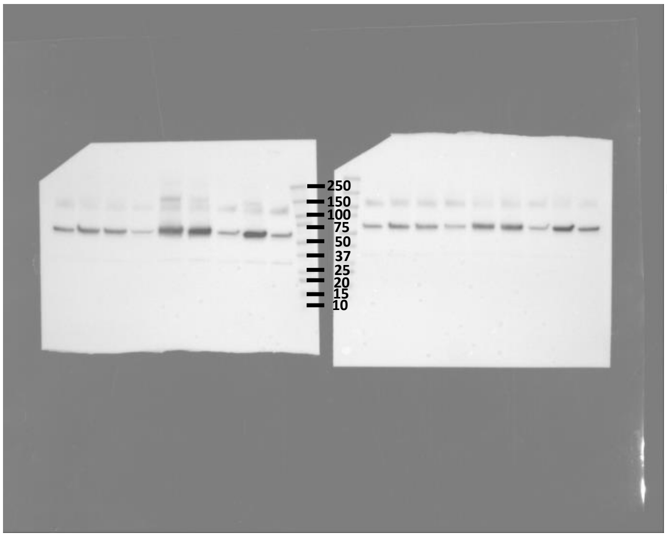

LAMP2 2023

LAMP2
100-130 kDa

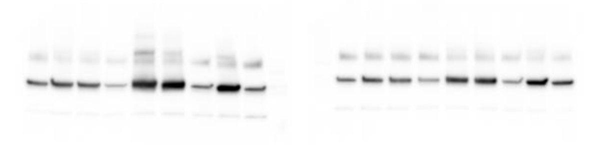

β-actin Batch 2023

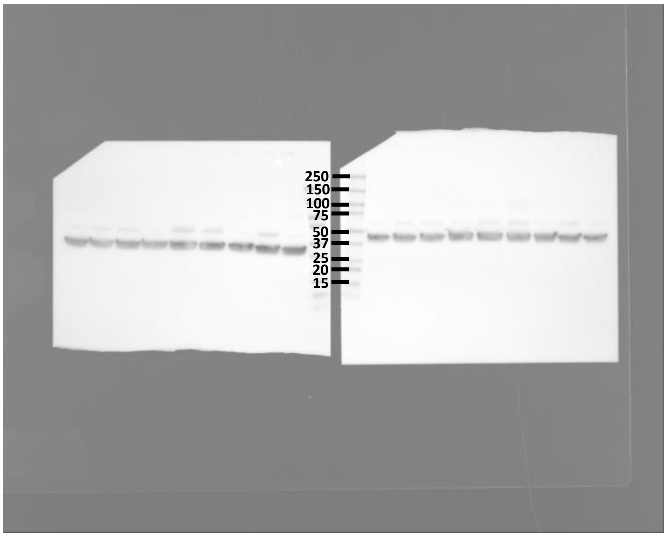

β-actin Batch 2023

β-actin
45 kDa

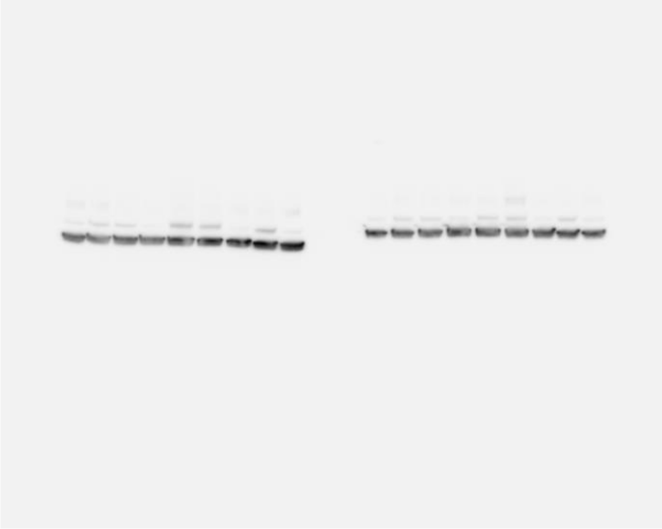

Blots for LC3A/B I/II and P-NF-κB expression quantification after Aβ42 treatment (Figure 4E, M) Batch 2023

Merge with ladder images

LC3A/B I/II Batch 2023, APOE4

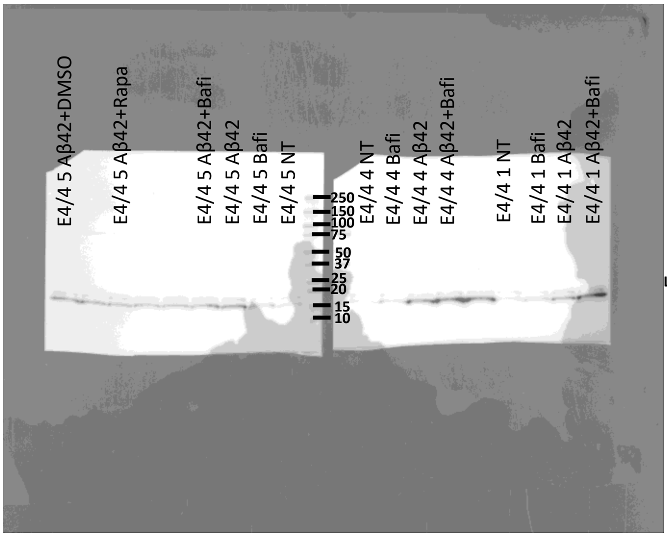

LC3A/B I/II
14, 16 kDa

Images for quantification

LC3A/B I/II Batch 2023

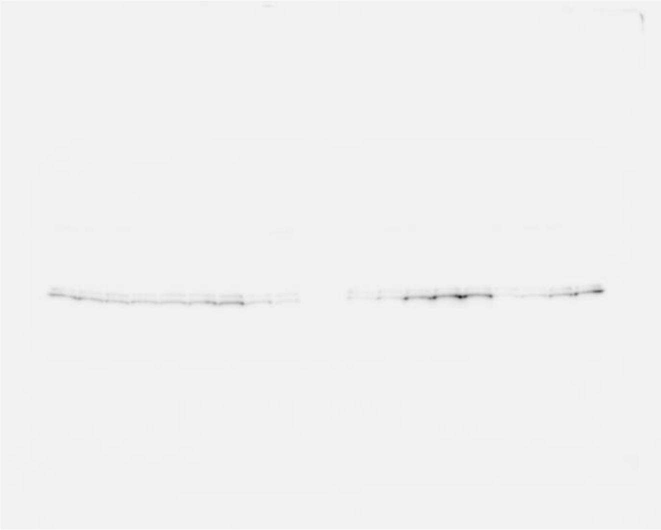

P-NF-κB Batch 2023

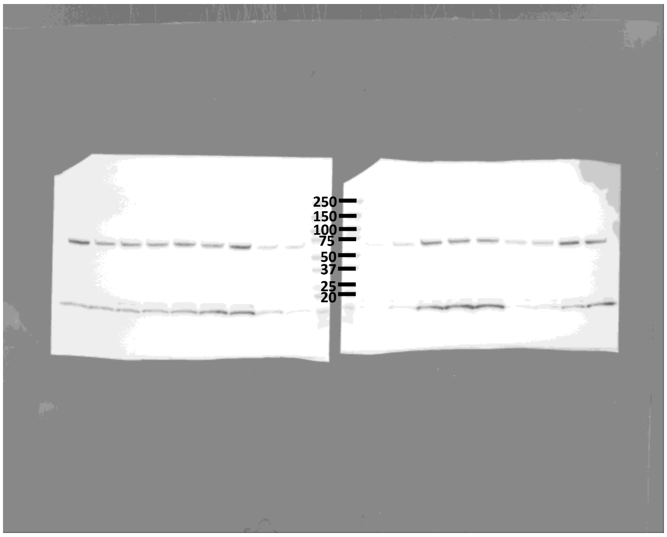

P-NF-κB
65kDa

P-NF-κB Batch 2023

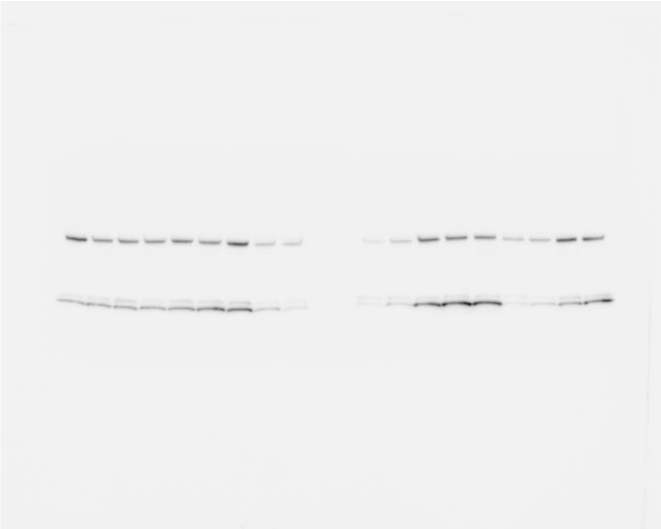

Blots for NF-κB and β-actin expression quantification after Aβ42 treatment (Figure 4 E, M) Batch 2023

Merge with ladder images

Images for quantification

NF-κB Batch 2023

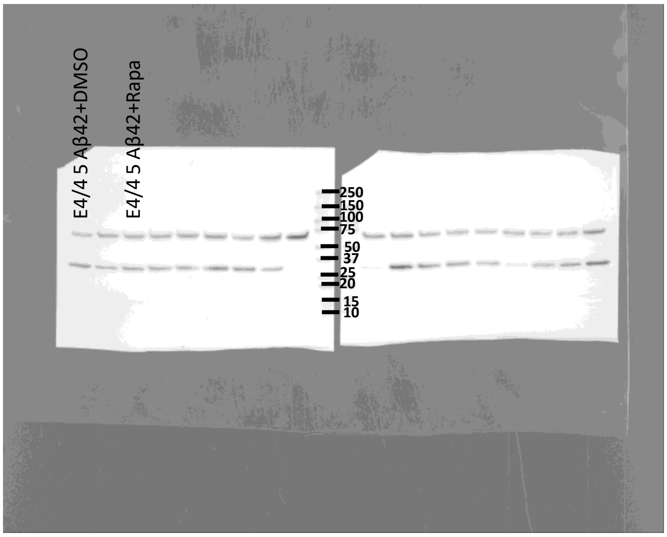

NF-κB Batch 2023

NF-κB
65kDa

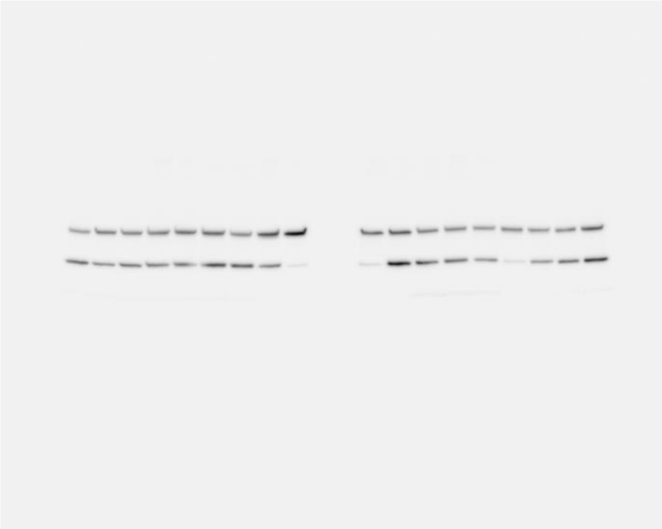

β-actin Batch 2023

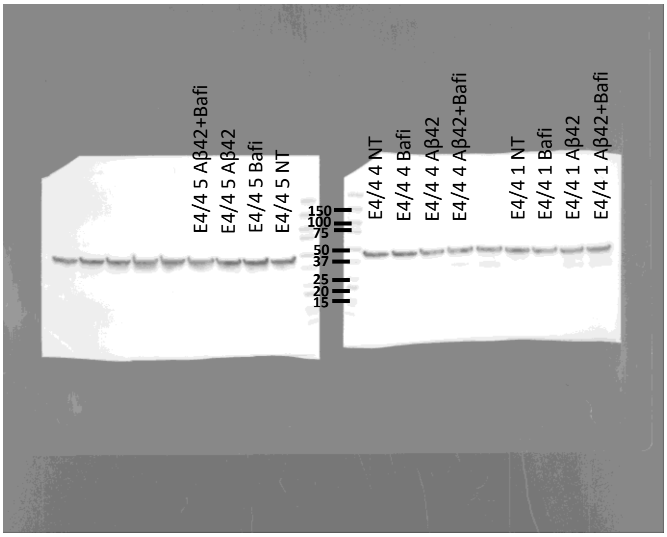

β-actin Batch 2023

β-actin
45 kDa

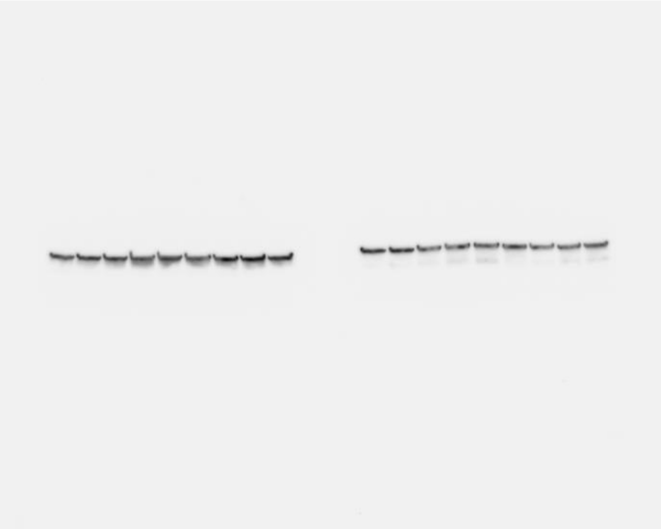

Blots for LC3A/B I/II and P-NF-κB expression quantification after Aβ42 treatment (Figure 4E) Batch 2023

Merge with ladder images

LC3A/B I/II Batch 2023, APOE3

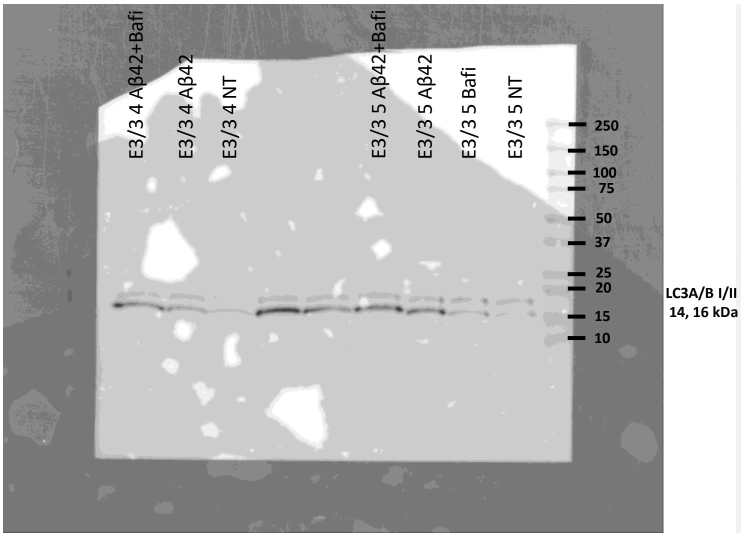

P-NF-κB Batch 2023

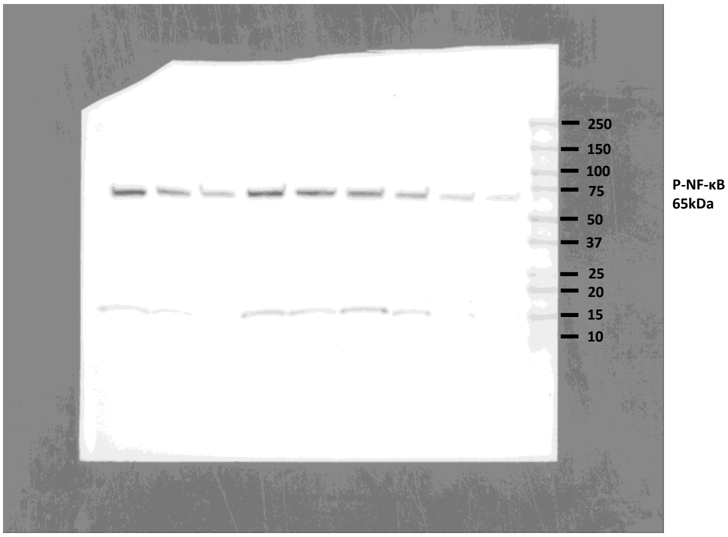

β-actin Batch 2023

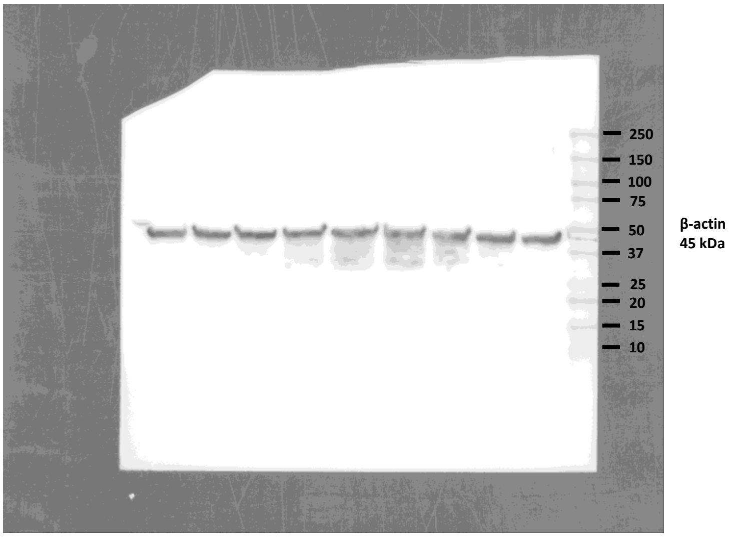

Images for quantification

LC3A/B I/II Batch 2023

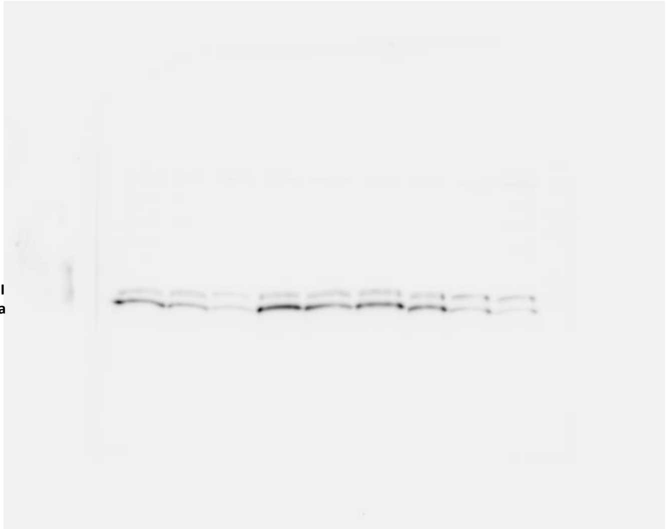

P-NF-κB Batch 2023

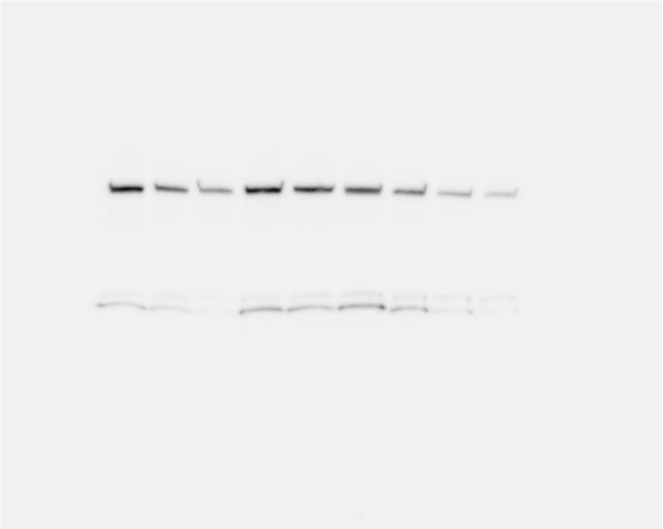

β-actin Batch 2023

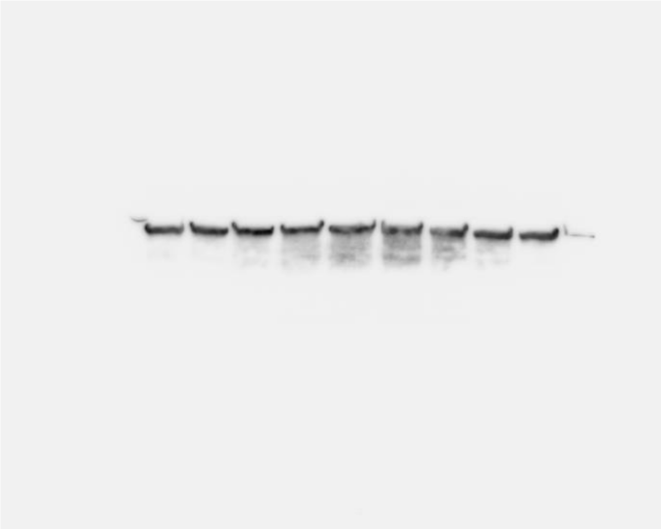

Blots for P-NF-κB p65 and P-S6 expression quantification (Figure 4 K, M) Batch 2023

Merge with ladder images

Images for quantification

P-S6 Batch 2023

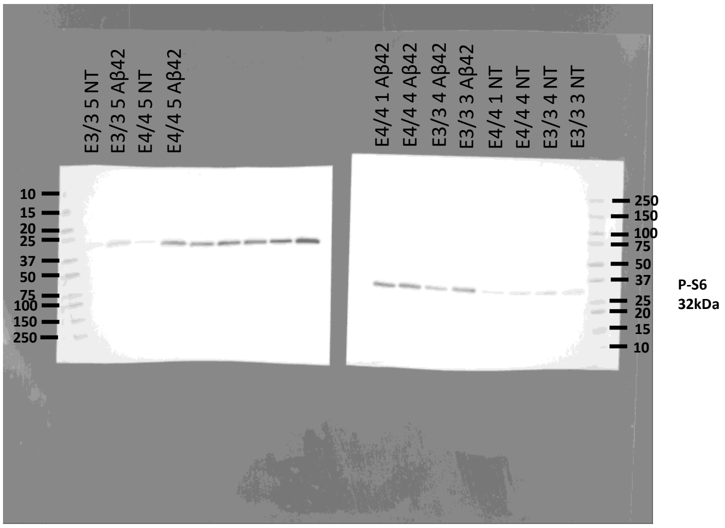

P-S6 Batch 2023

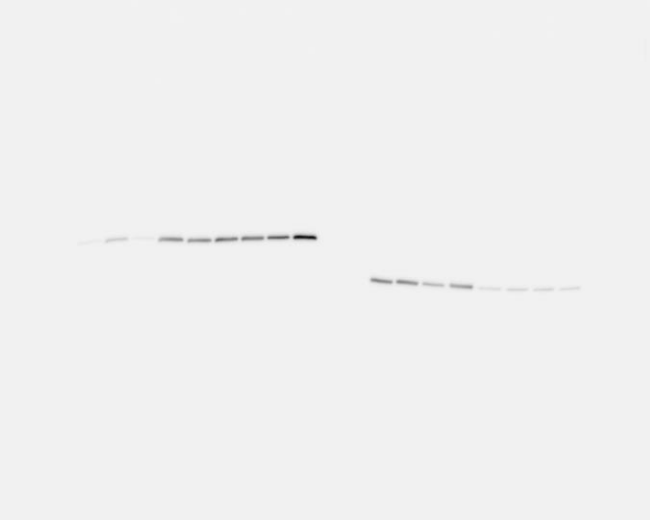

P-NF-κB p65 Batch 2023

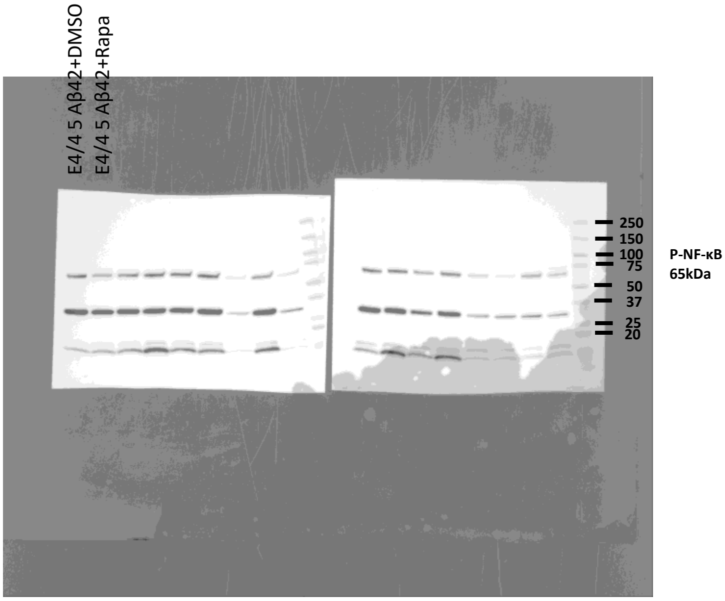

P-NF-κB p65 Batch 2023

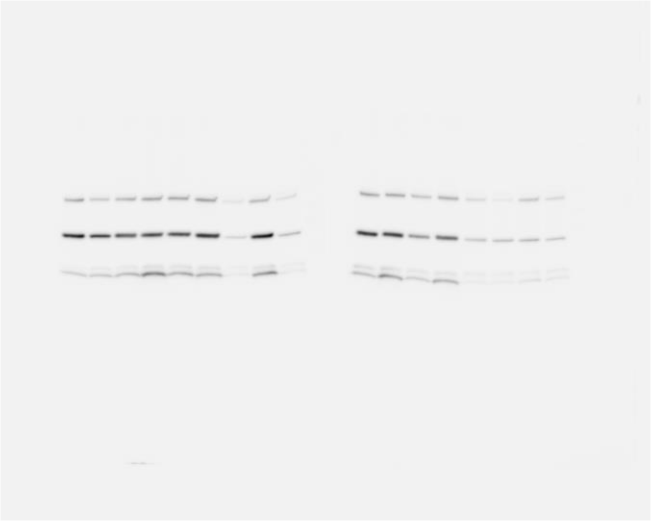

Blots for NF-κB p65 and β-actin expression quantification (Figure 4 K, M) Batch 2023

Merge with ladder images

NF-κB p65 Batch 2023

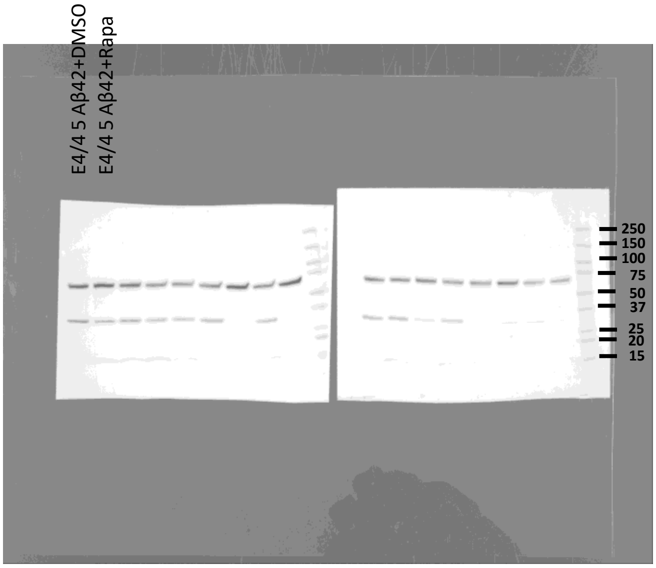

Images for quantification

NF-κB p65 Batch 2023

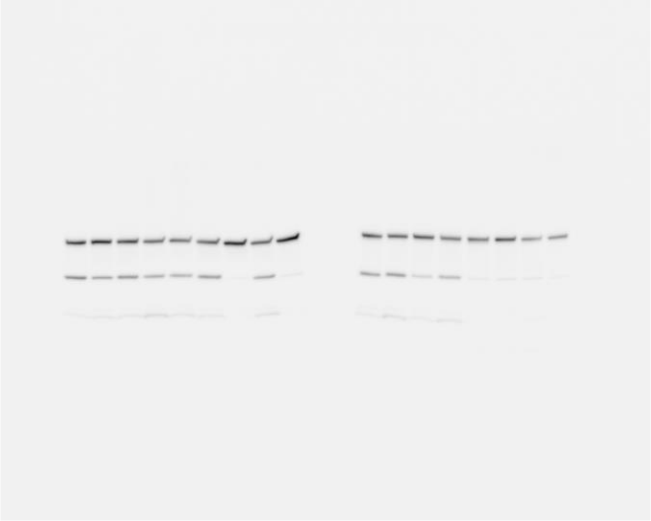

NF-κB
65kDa

β-actin Batch 2023

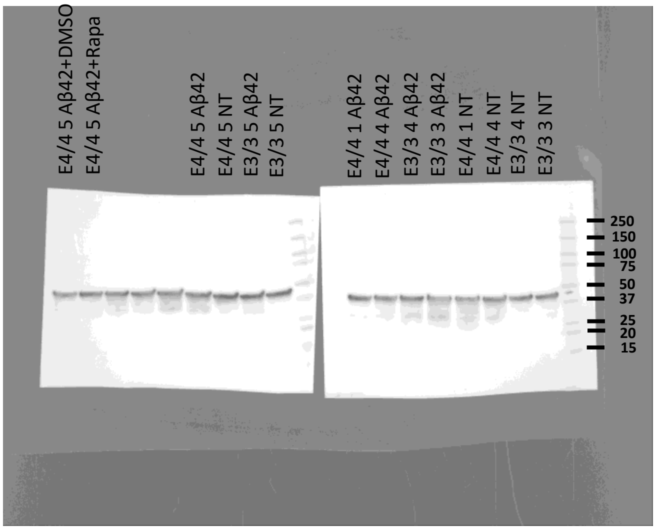

β-actin Batch 2023

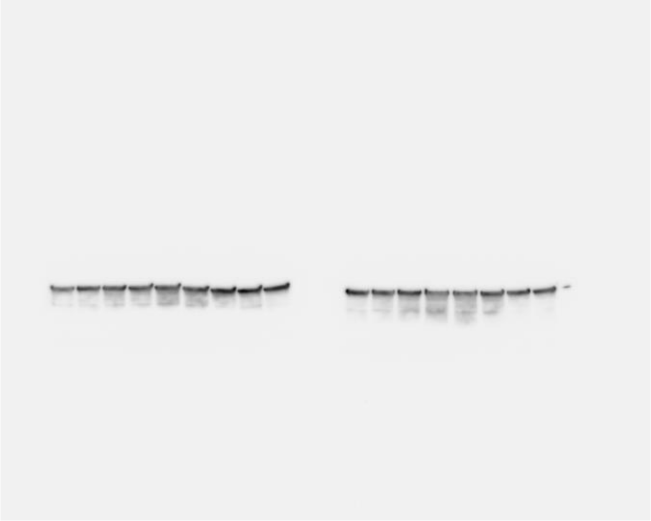

β-actin
45kDa
